# A review of the elusive bicolored iris Snouted Treefrogs (Anura: Hylidae:*Scinax uruguayus* group)

**DOI:** 10.1371/journal.pone.0222131

**Published:** 2019-09-25

**Authors:** Diego Baldo, Katyuscia Araujo-Vieira, Dario Cardozo, Claudio Borteiro, Fernando Leal, Martín O. Pereyra, Francisco Kolenc, Mariana L. Lyra, Paulo C. A. Garcia, Célio F. B. Haddad, Julián Faivovich

**Affiliations:** 1 Laboratório de Genética Evolutiva, Instituto de Biología Subtropical “Claudio Juan Bidau” (CONICET-UNaM), Posadas, Misiones, Argentina; 2 División Herpetología, Museo Argentino de Ciencias Naturales “Bernardino Rivadavia”—CONICET, Buenos Aires, Argentina; 3 Sección Herpetología, Museo Nacional de Historia Natural, Montevideo, Uruguay; 4 Laboratório de Herpetologia, Departamento de Zoologia, Instituto de Ciências Biológicas, Universidade Federal de Minas Gerais, Belo Horizonte, Minas Gerais, Brazil; 5 Departamento de Zoologia and Centro de Aquicultura (CAUNESP), Instituto de Biociências, Universidade Estadual Paulista, Rio Claro, São Paulo, Brazil; 6 Departamento de Biodiversidad y Biología Experimental, Facultad de Ciencias Exactas y Naturales, Universidad de Buenos Aires, Buenos Aires, Argentina; Fundacion Miguel Lillo, ARGENTINA

## Abstract

The genus *Scinax* currently includes more than 120 species, recovered in two major clades, the *S*. *catharinae* and the *S*. *ruber* clades. The latter comprises 75 species, most of which remain unassigned to any species groups, while 12 are included in the *S*. *rostratus* and *S*. *uruguayus* groups. In this paper we present a taxonomic review of the two species currently included in the *S*. *uruguayus* group, discussing some putative phenotypic synapomorphies of this group. Although *S*. *pinima* and *S*. *uruguayus* have been considered as distinct species, this has been based on scant evidence, and several authors doubted of their distinctiveness. Our study of available specimens of *S*. *pinima* and *S*. *uruguayus* corroborates that both are valid and diagnosable species based on phenotypic evidence. Furthermore, our results show that *S*. *pinima* previously known only from its type locality, has a much widespread distribution than previously thought (including the Brazilian states of Paraná, Santa Catarina, and Rio Grande do Sul), which, added to the biological information presented here allows to suggest the removal of this species from the “Data Deficient” IUCN Red List category to “Least Concern”. Also, we describe a new species formerly reported as *S*. aff. *pinima* and *S*. *uruguayus* from NE Argentina and some localities from the Brazilian State of Rio Grande do Sul. All species are diagnosed and characterized using adult and larval morphology, osteology, vocalizations, cytogenetics, and natural history.

## Introduction

The Neotropical genus *Scinax* Wagler, 1830 is the most species-rich in the family Hylidae with 124 species widely distributed from southern Mexico to central-eastern Argentina, Trinidad and Tobago, and St. Lucia [1]. The genus comprises two large clades, the *S*. *catharinae* and the *S*. *ruber* clades [2,3]. The latter includes 75 species, of which 12 are included in the two monophyletic species groups currently recognized, the *S*. *rostratus* and the *S*. *uruguayus* groups [3,4].

The *Scinax uruguayus* group was first recognized as the *Hyla uruguaya* group by Faivovich et al. [3] based on three putative morphological synapomorphies, the presence of bicolored iris in adults, enlargement of the posterior oral disc marginal papillae of larvae with respect to the lateral papillae (this was mistakenly inverted on pg, 40 and then corrected on pg, 97 in [3]), and the presence of two keratinized and dark colored plates on the sides of the lower jaw sheath [5,6]. In their phylogenetic analysis of Hylidae, Faivovich et al. [3] recovered their single exemplar of the group, *H*. *uruguaya* Schmidt, 1944 as the sister taxon of the *Scinax ruber* clade. These authors, consequently, transferred the *H*. *uruguaya* group to the genus *Scinax* in order to remedy its paraphyly. This result corroborated the proposal by Kolenc et al. [6] regarding a possible relationship of the former *Hyla pinima* Bokermann and Sazima, 1973 and *H*. *uruguaya* with the *Scinax ruber* clade (sensu [2]) as the vent tube in tadpoles of these taxa does not reach the free margin of the lower fin, which is to date the single morphological synapomorphy known for the referred clade [2].

Since the results of Faivovich et al. [3], the *Scinax uruguayus* group was repeatedly recovered as the sister taxon of the remaining species of the *S*. *ruber* clade in subsequent phylogenetic analyses, being *S*. *pinima* (as *S*. *uruguayus* CFBH 5788; see S1 Appendix) the only exemplar species of the group included in these analyses [3,7–10]. Two morphological character states are considered putative synapomorphies of the *S*. *uruguayus* group: (i) bicolored iris in adults and (ii) presence of two keratinized and dark colored plates on the sides of the lower jaw sheath in larvae. The reduction of toe webbing and marginal papillae on the posterior margin of the oral disc larger than those on the lateral margins in larvae could be other synapomorphies of this group [3,6].

*Scinax uruguayus* occurs in Uruguay, south Brazil, and one locality in northeastern Argentina, whereas *S*. *pinima* is known only from its type locality and nearby farther north in central-eastern Brazil (e.g., [5,6,11–14]). *Scinax uruguayus* was described as *Hyla uruguaya* by Schmidt [15], from Quebrada de los Cuervos, Departamento de Treinta y Tres, Uruguay. It was subsequently considered a junior synonym of *Hyla minuta* (Barrio 1967), until Langone [16] resurrected it as a valid species. Kolenc et al. [6] provided a natural history account on Uruguayan populations. *Scinax pinima* was described as *Hyla pinima* by Bokermann and Sazima [5], from Serra do Cipó, Jaboticatubas, State of Minas Gerais, and no relevant information about its biology was published since then.

Several authors raised doubts about the validity of *Scinax pinima* and suggested that it could be a junior synonym of *S*. *uruguayus*, given their morphological resemblance [6,11,13,17]. Two reasons that have been considered to recognize them as distinct species are (i) the geographic distance of ~1,200 km between the only known population of *S*. *pinima* in Serra do Cipó, and the northernmost known record of *S*. *uruguayus* in the Municipality of Palma, State of Paraná, Brazil [5,18]; and (ii) the coloration of the head: presence of a white V-shaped dorsal blotch in *S*. *pinima*, yellow cream in *S*. *uruguayus* [5,6,16,17].

The goal of this study is to review the taxonomy of the *Scinax uruguayus* group on the basis of new and existing information about external morphology, osteology, bioacoustics, cytogenetics, geographical distribution, and natural history. We also describe a new species of this group from northeastern Argentina and southeastern Brazil, and discuss the taxonomic distribution of the putative morphological synapomorphies that have been proposed for the group.

## Materials and methods

Examined adults, juveniles, and tadpoles, including types of *Scinax pinima* and *S*. *uruguayus*, are listed in the S1 Appendix. Specimens collected for this study were euthanized with lidocaine, fixed in 10% formalin, and stored in 70% ethanol (adults and juveniles) or 10% formalin (tadpoles). Institutional abbreviations follow Sabaj [19].

### Adult external morphology

Dorsal and profile outline standards of snout shape follow Heyer [20]. Ten measurements (in millimeters) were taken with digital calipers (± 0.01 mm), after Duellman [21]: snout-vent length (SVL), head length (HL), head width (HW), internarial distance (IND), interocular distance (IOD), eye diameter (ED), eye-nostril distance (END), tympanum diameter (TD), tibia length (TL), and foot length (FL). We also considered additional measurements: third finger disk diameter (3FD) and fourth toe disk diameter (4TD) employed by Napoli and Caramaschi [22]. Webbing formula follows Savage and Heyer [23] as modified by Myers and Duellman [24]. Fingers were numbered II to V following Fabrezi and Alberch [25]. Terminology of nuptial pad morphology is that proposed by Luna et al. [26]. Sex was determined by visual inspection of external secondary sexual characters (nuptial pads, vocal slits, and expansion of the vocal sac) or the gonads by dissection.

### Adult skeletal morphology

Osteological descriptions are based on adult male specimens that were cleared and double stained with alcian blue and alizarin red [27]. The terminology of skull and postcranium follows Jurgens [28] and Trueb [29,30]; Alberch and Gale [31] for phalangeal formulae; Fabrezi [32,33] and Fabrezi and Alberch [34] for carpal and tarsal elements; and Trewavas [35] and Faivovich [2] for larynx.

### Larval external morphology

Terminology follows Altig and McDiarmid [36], excepting the position of intestinal mass, which follows Faivovich [2]. Measurements (± 0.1 mm) were based on 12 tadpoles of the new species at stages 35–37, 12 tadpoles of *Scinax pinima* at stages 28–30 and 37, and 15 tadpoles of *S*. *uruguayus* at stages 31–33 (stages according Gosner [37]). All but total length that was measured with digital calipers, were taken with an ocular micrometer under a stereoscopic microscope Leica MZ6. Eight of them follow Altig and McDiarmid [36]: total length (TL), body length (BL), tail length (TAL), tail fin height (FH), tail muscle height (TMH), tail muscle width (TMW), interorbital distance (IOD), and internarial distance (IND). Eleven follow Lavilla and Scrocchi [38]: body maximum height (BMH), body maximum width (BMW), body width at nostrils (BWN), body width at eye level (BWE), eye diameter (ED), rostro-spiracular distance (RSD), eye-nostril distance (END), fronto-nasal distance (FN), nostril diameter (ND), oral disc width (ODW), and dorsal gap length (DG). Methylene blue was employed to enhance visualization of oral disc structures.

### Buccopharyngeal morphology of larvae

One tadpole of *Scinax pinima* (stage 28, UFMG 2262) was dissected as done by Wassersug [39] to expose the buccopharyngeal cavity, that was described following the standards and terminology of Wassersug [39,40].

### Recording and advertisement call analyses

Advertisement calls analyzed in this study are deposited in the Colección Bioacústica del Laboratorio de Genética Evolutiva (LGE-B) and Coleção Bioacústica da Universidade Federal de Minas Gerais (CBUFMG). Calls were recorded with diverse devices including tape (Marantz PMD-222, Roland R-05 Studio, Sony WM-D6C) and digital recorders (Marantz PMD-660), equipped with a Sennheiser ME66/K6 or ECM–MS907 directional microphones. Tape recordings were digitized with a sampling rate of 44.1 kiloHertz (kHz) and 16-bit resolution. Calls were analyzed with the software Raven Pro v1.5 (Bioacoustics Research Program 2014). Spectrograms were generated with window type Hann, window size = 512 samples, overlap = 70%, hop size = 3.49 ms, DFT size = 1024 samples, and grid spacing = 43.1 kHz. Sound graphics were obtained using Seewave [41] package of R platform (R Core Team 2014), using Hanning window, FFT = 512, and 50% overlap. The acoustic characterization follows the format proposed by Köhler et al. [42]. Temporal parameters such as note duration, interval between notes, note rate (notes per second), and pulses per note and pulse rate (pulses per second) are those defined by Cocroft and Ryan [43]. Temporal parameters were measured from oscillograms and spectral parameters from spectrograms. Dominant frequency was obtained using the function “Peak frequency” from Raven Pro v1.5.

### Cytogenetics

Chromosome spreads were prepared from intestinal epithelium and testes [44]. Cellular spreads were stained with a Giemsa-PBS solution (pH 6.8). The silver-staining of nucleolar organizer regions (*Ag-NORs*) and C-banding techniques were performed according to Howell and Black [45] and Sumner [46], respectively. The relative length (RL), centromeric index (CI), and centromeric ratio (CR) were scored using the software Micromeasure v3.3 [47]. Karyotypes were arranged according to decreasing chromosome size following the terminology of Green and Sessions [48,49]. We used *x* (basic chromosome number), *2n* (somatic chromosome number), and *FN* (fundamental number of chromosome arms) as suggested by White [50]. Other abbreviations are: *p* (short arm); *q* (long arm); *sc* (secondary constrictions).

### Nomenclatural acts

The electronic edition of this article conforms to the requirements of the amended International Code of Zoological Nomenclature, and hence the new names contained herein are available under that Code from the electronic edition of this article. This published work and the nomenclatural acts it contains have been registered in ZooBank, the online registration system for the ICZN. The ZooBank LSIDs (Life Science Identifiers) can be resolved and the associated information viewed through any standard web browser by appending the LSID to the prefix “http://zoobank.org/”. The LSID for this publication is: urn:lsid:zoobank.org:pub: C0C1C6B7-0FAB-4EF6-A3BE-D61B9FBA0FEB. The electronic edition of this work was published in a journal with an ISSN, and has been archived and is available from the following digital repositories: PubMed Central and LOCKSS.

## Results

The study of the available specimens of *Scinax pinima* and *S*. *uruguayus*, including types and topotypic material (S1 Appendix), corroborates that *S*. *pinima* and *S*. *uruguayus* are valid, clearly diagnosable species. In addition, populations from Argentina and some localities from the Brazilian State of Rio Grande do Sul, previously reported as *S*. aff. *pinima* and *S*. *uruguayus* [14,51–56] actually belong to a new species that is described below.

### Taxonomic accounts

#### *Scinax fontanarrosai* sp. n

urn:lsid:zoobank.org:act:39C933EB-7EDF-4AEC-9B52-F068FAA33047.

*Hyla uruguaya*—*non* Schmidt [15]. Giraudo et al. [14], *partim*.

*Scinax uruguayus*—Leite et al. [51], *partim*. Kwet et al. [52], *partim*. Vaira et al. [54]. Zaracho et al. [55]. Marin da Fonte et al. [56], *partim*.

*Scinax* aff. *pinima*—Alcalde et al. [53].

*Julianus uruguayus*—Duellman et al. [9], *partim*. Ferrão et al. [57], *partim*.

#### Holotype

LGE 4451, adult male, from Argentina, Misiones, Departamento Capital, Ruta Nacional 12, km 1329 (27.443694° S, 56.028611° W; 146 m above sea level, a.s.l.), collected on 21 October 2005 by D Baldo, C Borteiro, D Cardozo, and F Kolenc.

#### Paratypes

All males unless otherwise stated. Forty-two adults collected at the type locality: MACN 53292–94, LGE 4452−4461, 4463–74, 4477–84, 4485 (female), 9606−8, 11901−2, 20538−9, and 22088. Thirty-seven adults collected in nine localities in the Provinces of Corrientes and Misiones, Argentina: LGE 2041−3 and 4804 from Estancia Santo Domingo (27.683333° S, 56.133333° W), Departamento Ituzaingó, Corrientes. MACN 53295, LGE 4380, 4382, 4387, 4388 (female), and 4389 from Ruta Nacional 12, 2.6 km NE from San Borjita (27.477167° S, 56.073861° W), Departamento Ituzaingó, Corrientes. LGE 4383−6 from Ruta Nacional 14, 5 km from Gobernador Virasoro (28.000000° S, 56.016667° W), Departamento Santo Tomé, Corrientes. LGE 94−7 from Ruta Provincial 2, 6.5 km W from Santa María (28.000000° S, 56.016667° W), Departamento Concepción, Misiones. LGE 98−9 from 3.5 km S Itacaruaré (27.902533° S, 55.273889° W), Departamento San Javier, Misiones. LGE 3719 from Barrio Santa Helena, Garupá (27.464508° S, 55.888472° W), Departamento Capital, Misiones. LGE 6016−9 from Ruta Provincial 1, 5.6 km NE from Azara (28.011667° S, 55.714444° W), Departamento Apóstoles, Misiones. LGE 6388−90 from Ruta Nacional 12, 8.5 km NW from intersection with Ruta Nacional 14 (27.914444° S, 56.066667° W), Departamento Ituzaingó, Corrientes. LGE 9609−10 from Ruta Nacional 12, Arco-Garita, km 1330, Posadas (27.457778° S, 56.009722° W), Departamento Capital, Misiones. LGE 9611−7 from Ruta Nacional N 12, Arco-Garita, km 1332, Posadas (27.462222° S, 55.991111° W). All specimens were collected at different dates on October and November 2004, October 2005, September 2007, December 2014, December 2015, and January 2018 by D Baldo, C Borteiro, D Cardozo, F Kolenc, MO Pereyra, Y Alippe, M Boeris, L Cotichelli, JM Ferro, S Nenda, and C Tomatis.

#### Referred specimens

LGE 4486−90, cleared and double stained adult males, collected at the type locality. LGE 12, 8529, and 10392, larval lots, collected at the type locality. LGE 49–56, juveniles, collected at the type locality. LGE 2040, cleared and double stained adult male, from Estancia Santo Domingo, Departamento Ituzaingó, Corrientes. UFRGS 3328, adult male, from Fazenda São Francisco, Stora Enso, Alegrete, Rio Grande do Sul, Brazil. UFRGS 4442 and 4465–75, adult males, from Corredor dos Keller, 1° Distrito, Manoel Viana, Rio Grande do Sul, Brazil.

#### Diagnosis

The new species is assigned to the *Scinax uruguayus* group of the *S*. *ruber* clade based on the presence of bicolored iris in adults and two keratinized and dark colored plates on the sides of the lower jaw sheath in larvae; the two known putative synapomorphies of this group [3,6]. Additionally, it could be diagnosed by the following set of characters: (1) small size in females (SVL 24.1–24.2 mm; *n* = 2); (2) head sub-elliptical in dorsal view; (3) presence of two or three poorly distinguishable interorbital grooves; (4) anterior portion of the choanae not concealed by the palatal shelf of the maxillary arch when roof of mouth is viewed from below; (5) V-shaped cephalic blotch; (6) bicolored iris with a golden upper half and a dark brown to black lower half; (7) discs of the fingers and toes gray to dark brown in life; (8) hidden surfaces of thighs and tibia orange in life; (9) frontoparietals juxtaposed or slightly separated, almost completely concealing fontanelle; (10) laminar dentigerous process of the vomers without teeth; (11) palatines reduced to thin slivers; (12) intercalary elements between ultimate and penultimate phalanges partially mineralized; (13) larynx with oval arytenoids, which have a slight medial constriction in dorsal view; (14) advertisement call composed of a single, short (49–66 ms), and pulsed note (25–31 pulses/note), emitted at a rate of 3.9–4.9 notes/s; (15) pulse rate of 490–540 pulses/s; (16) notes with pulses that are increasingly modulated for the first quarter of the note, remaining with relatively constant amplitude in the second quarter, and then decrease up to the end; (17) highly pitched advertisement call, with harmonic structure; and (18) dominant frequency between 5513–6159 Hz.

#### Comparisons with *Scinax pinima* and *S*. *uruguayus*

*Scinax fontanarrosai* sp. n. differs from *S*. *pinima* (character states in parentheses) as females are smaller, SVL 24.1–24.2 mm (29.0 mm in the only known female; [5]); sub-elliptical head in dorsal view (broadly rounded); two or three poorly distinguishable interorbital grooves (three grooves fairly evident in all topotype specimens and poorly marked in specimens of southern populations); bicolored iris with a golden upper half and dark brown to black lower half (golden upper half and dark brown lower half with scattered small, round, and golden chromatophores); discs of fingers and toes gray to dark brown in life (bicolored discs of fingers with a gray proximal half and a light brown to orange distal half); and hidden surfaces of thighs and tibia orange in life (light purple).

The new species also differs from *Scinax pinima* by having frontoparietals juxtaposed or slightly separated resulting in an almost completely concealed fontanelle (frontoparietals as slender strips at the level of the fontanelle resulting in an almost completely exposed fontanelle); laminar dentigerous process of the vomers without teeth (pointed dentigerous process without teeth); and larynx with oval arytenoids, which have a slight medial constriction in dorsal view (arytenoids oval, without medial constriction in dorsal view).

The advertisement call of *Scinax fontanarrosai* sp. n. differs from that of *S*. *pinima* as notes are longer ranging between 49–66 ms (30–43 ms); and composed by 25–31 pulses (11–14 pulses) that are increasingly modulated for the first quarter of the note, being relatively constant in amplitude in the second quarter, and then decreasing towards the end (notes with the first pulse distinctly lower than the second, and decreasing amplitude modulation from the second pulse to the last one); notes emitted at a rate of 3.9–4.9 notes/s (3.0–3.3 notes/s); pulse rate of 490–540 pulses/s (275–355 pulses/s); notes having harmonic structure (harmonic structure absent); and dominant frequency ranging between 5513–6159 Hz (3919–4479 Hz).

*Scinax fontanarrosai* sp. n. can be differentiated from *S*. *uruguayus* (character states in parentheses) by the sub-elliptical head in dorsal view (broadly rounded); presence of two or three poorly distinguishable interorbital grooves (grooves absent); anterior portion of the choanae not concealed by the palatal shelf of the maxillary arch when roof of mouth is viewed from below (anterior portion of the choanae concealed by the palatal shelf of the maxillary arch when roof of mouth is viewed from below); V-shaped cephalic blotch (subtriangular cephalic blotch); bicolored iris with a golden upper half and dark brown to black lower half (iridescent golden upper half and golden lower half with brown reticulations); discs of fingers and toes gray to dark brown in life (golden yellow to orange); and hidden surfaces of thighs and anterior surface of tibia orange in life (light purple).

Furthermore, the new species differs from *Scinax uruguayus* by the occurrence of juxtaposed or slightly separated frontoparietals, resulting in an almost completely concealed fontanelle (frontoparietals as slender strips at the level of the fontanelle resulting in an almost completely exposed fontanelle); laminar dentigerous process of the vomers without teeth (thick, rectangular dentigerous process with 3–4 teeth); intercalary elements between ultimate and penultimate phalanges partially mineralized (completely mineralized); and larynx with oval arytenoids, which have a slight medial constriction in dorsal view (arytenoids oval, without medial constriction in dorsal view).

The advertisement call of *Scinax fontanarrosai* sp. n. differs from that of *S*. *uruguayus* as notes are also longer, ranging from 49–66 ms (17–28 ms), with higher number of pulses, 25–31 (7–10 pulses), and distinct modulation (notes with pulses of decreasing amplitude from the first to the last one); pulse rate of 490–540 pulses/s (286–476 pulses/s); notes having harmonic structure (harmonic structure absent); and dominant frequency ranging between 5513–6159 Hz (3833–4651 Hz).

#### Description of holotype

**Fig 1:**
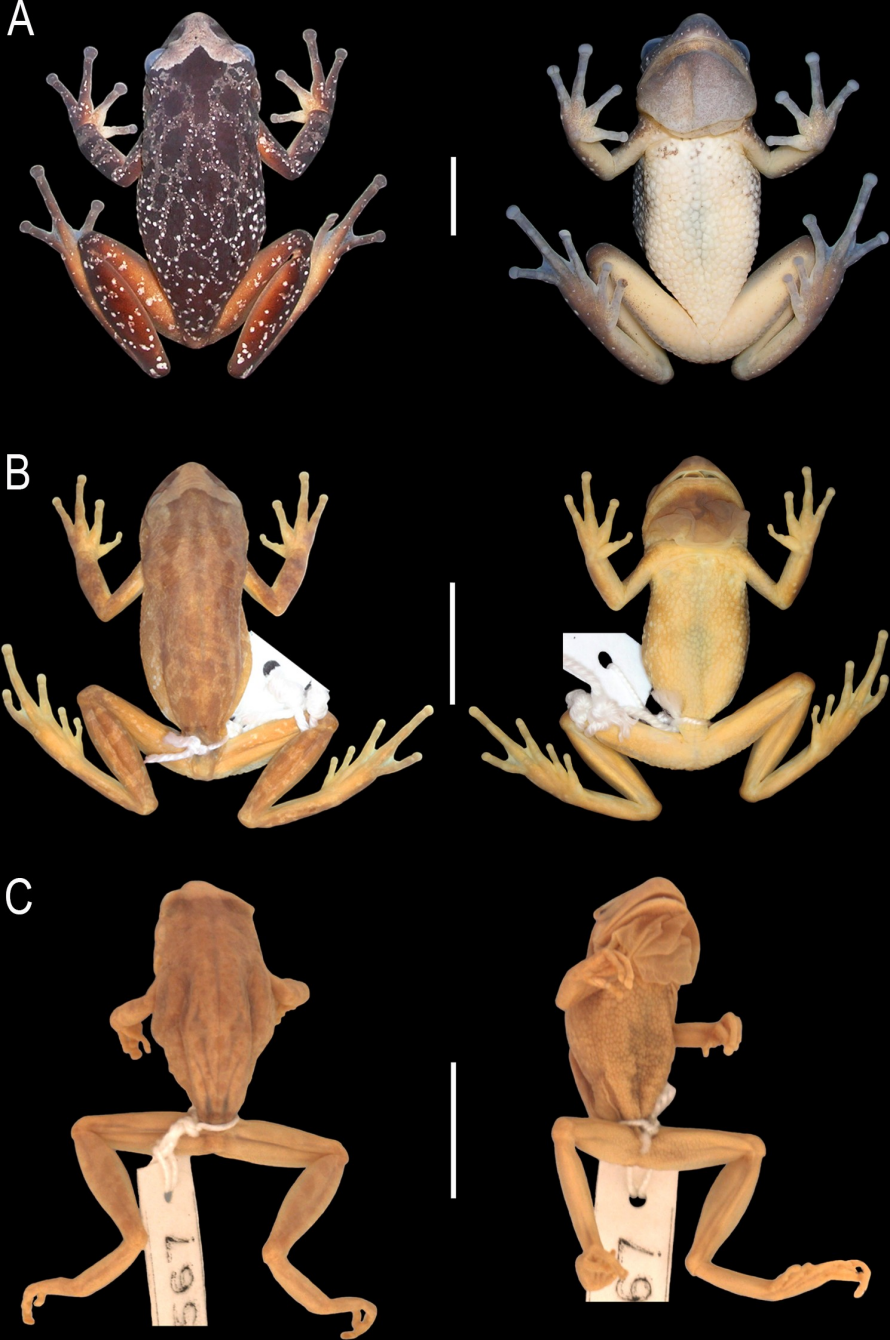
**Dorsal (left) and ventral (right) views of the body**. (A) *Scinax fontanarrosai* sp. n. (LGE 4451, holotype), (B) *S*. *pinima* (WCAB 46238, holotype; now MZUSP 73668), and (C) *S*. *uruguayus* (FMNH 10567, holotype). Scale bars = 50 mm.

**Fig 2:**
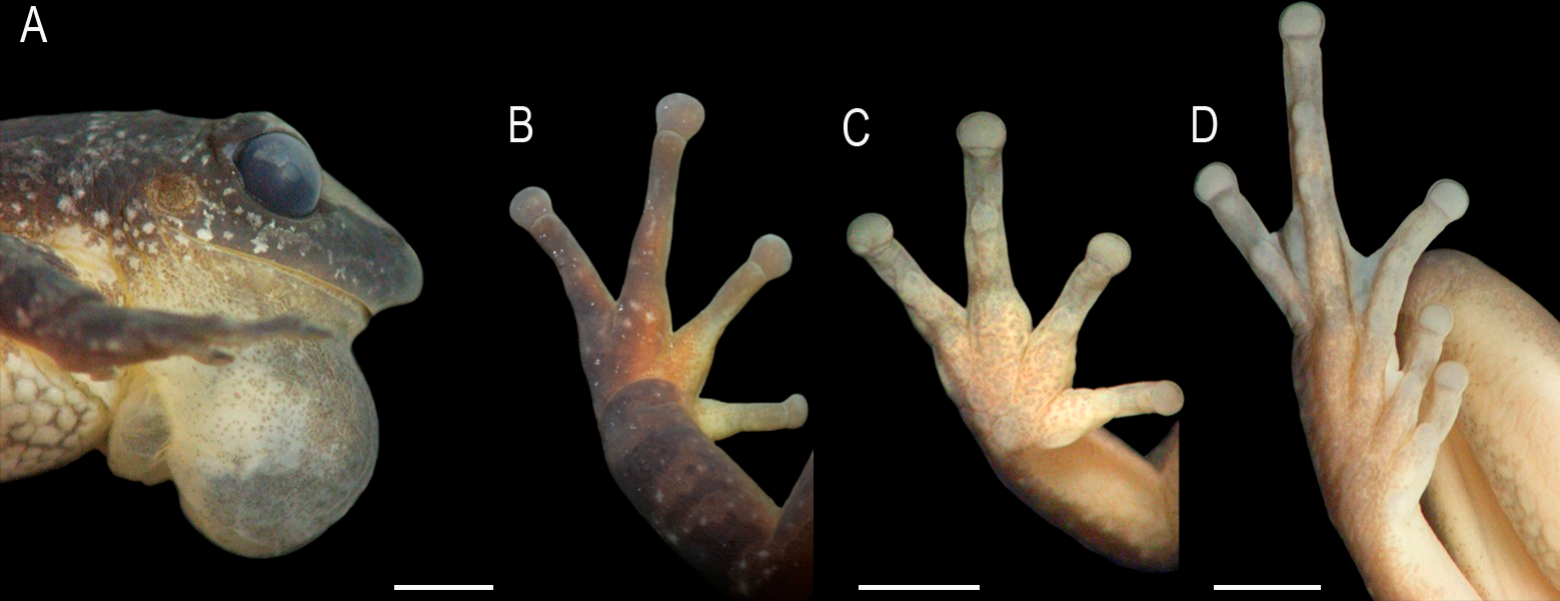
*Scinax fontanarrosai* sp. n., holotype (LGE 4451). (A) Head in lateral view. (B) Left hand in dorsal view. (C) Left hand in ventral view. (D) Left foot in ventral view. Scale bars = 2 mm.

**Fig 3:**
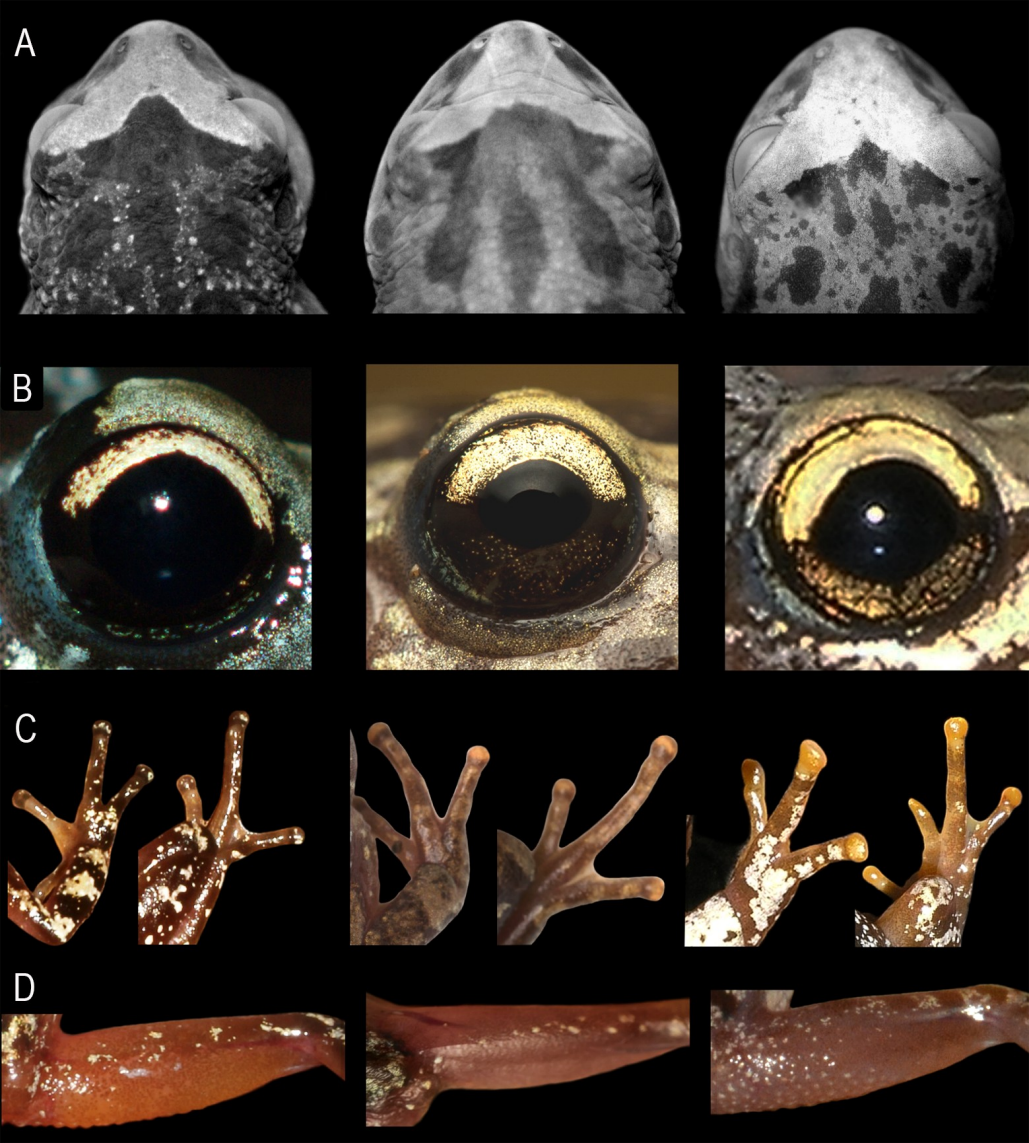
Adult external morphological characters. *Scinax fontanarrosai* sp. n. (left), *S*. *pinima* (central), and *S*. *uruguayus* (right). (A) Head in dorsal view. (B) Color pattern of the bicolored iris. (C) Dorsal views of right hand and foot showing the color pattern of the discs in life. (D) Color pattern of the posterior surface of thighs in life.

Body slightly robust (Fig 1A). Head length 29.2% SVL, slightly longer than wide (HW/HL = 0.97). Snout nearly rounded in dorsal view and protruding in lateral view (Fig 2A). Head sub-elliptical in dorsal view (Fig 3A). Nostril tear-shaped, slightly protruded, located nearer the tip of the snout than to the eye, and laterally oriented; distance between nostrils 82.6% of IOD. *Canthus rostralis* rounded. Loreal region concave, slightly constricted in dorsal view. Eye protuberant (ED 9.7% SVL), ED 40% larger than END, almost equal to IOD. Tympanum oval, with distinct tympanic annulus and tympanic membrane, separated from eye by a distance almost equal to TD. TD 57.1% of ED. Supratympanic fold poorly developed. Vocal sac single, median, subgular, well-developed, externally evident by the loose skin; longitudinal folds evident in deflated position. Vocal slits present, located diagonally to the longitudinal body axis, originating laterally to the tongue and running towards the corner of the mouth. Tongue ovoid, free laterally and posteriorly not notched. Choanae rounded, widely separated medially, not concealed by the palatal shelf of the maxillary arch in ventral view. Vomerine teeth absent; premaxillary and maxillary teeth present.

Arm slender, forearm robust. Axillary membrane absent. Ulnar tubercles absent (Fig 2B). Fingers slender; relative length II<V<III<IV. Subarticular tubercles single and rounded (Fig 2C). Metacarpal tubercles undifferentiated. Discs rounded; on Finger II 25% smaller than 3FD. Finger webbing basal. Nuptial pads present, light-colored, without macroscopically evident epidermal projections (Fig 2B). Hind limbs slender; TL 48.6% SVL; foot length 47.2% SVL. Toes slender; relative length I = II < V < III < IV. Subarticular tubercles single and rounded (Fig 2D). Outer metatarsal tubercle poorly developed and flat; inner metatarsal tubercle absent. Discs rounded. Toe webbing formula I 2^+^–2^+^ II 2^-^–3^+^ III 3^+^–2^-^ IV 3^+^–2^-^ V. Tarsal fold absent. Dorsal and ventral skin slightly granulated, with small warts sparse on the dorsal surface. Ventral region markedly granulated. Pectoral fold with only a preaxillar element. Cloacal opening at upper level of thighs; cloacal sheath absent.

#### Measurements of the holotype (mm)

SVL 21.6; HL 6.3; HW 6.1; IND 1.9; IOD 2.3; ED 2.1; END 1.5; TD 1.2; TL 10.5; FL 10.2; 3FD 1.0; 4FD 1.0.

#### Coloration of the holotype in life

Colors and pattern descriptions of the holotype in life are based on field notes. Head with a light brown V-shaped blotch, extending from the tip of the snout to the middle of upper eyelids. Dorsum dark brown-black with a light gray or white reticulated pattern and scattered, small, white spots. The dark brown-black background is defined as several ovoid to round dark blotches and a big rhomboidal black blotch behind the V-shaped cephalic blotch. Dorsal surface of arms dark brown with gray transverse bars. Hind limbs dark brown with small white spots. Hidden surfaces of thighs and tibia orange. Pectoral and abdominal regions whitish, with scattered dark spots; dark brown colored vocal sac. Ventral region of arms dark brown, bearing a few small white spots. Black horizontal elliptical pupil; bicolored iris, with upper half golden and the lower half dark brown.

#### Coloration of the holotype in preservative

Dorsum brown with gray blotches and bars; white spots maintained. Whitish coloration on the ventral regions maintained. Spots on pectoral and gular regions became dark gray. Orange coloration on the hidden surfaces of thighs and tibia maintained.

#### Etymology

The new species is named in honor to the writer and cartoonist Roberto “El Negro” Fontanarrosa (1944−2007), in recognition of his vast contribution to the Argentinean culture. His work always included elements of nature, like the amphibians.

#### Variation in type series

**Fig 4:**
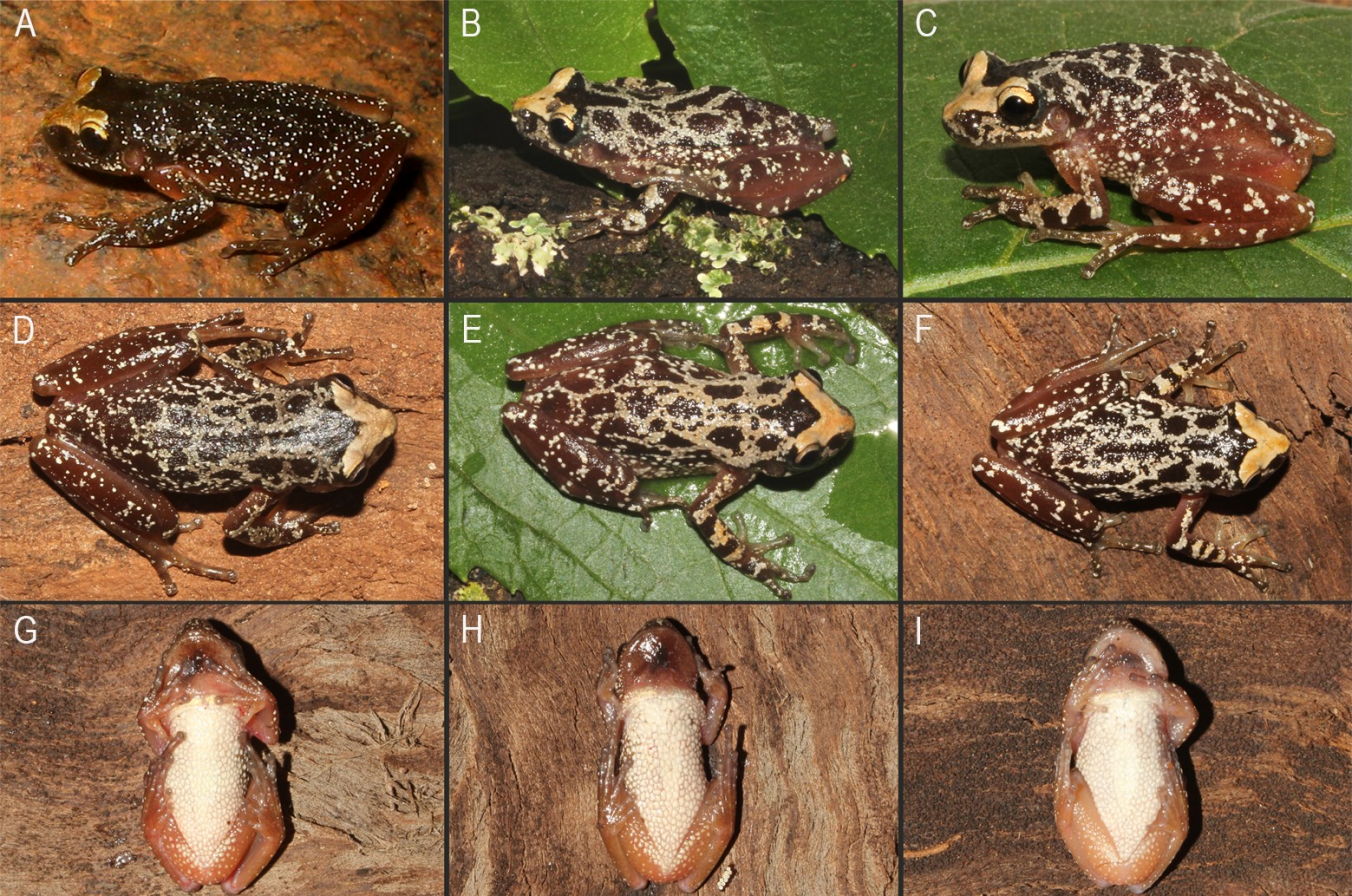
Color pattern of *Scinax fontanarrosai* sp. n. in life. (A–C) Dorsolateral, (D–F) dorsal, and (G–I) ventral views.

Measurements are presented in Table 1. Dorsal coloration varies in that the V-shaped cephalic blotch can be whitish or light brown, and the gray to white reticulation could extend to different areas, even covering some or most of the dorsal surface (Fig 4). Seven metamorphs have a similar coloration to that of adults, but with the gray to white reticulation somewhat lighter. Sexual dimorphism is evident: males bear vocal slits, quite expanded, dark brown colored vocal sacs, and light-colored nuptial pads without macroscopically evident epidermal projections. The two available females (SVL 24.1–24.2 mm) are slightly larger than males (SVL 19.1–23.3 mm, *n* = 78).

**Table 1:** Measurements (in mm) of adults. *Scinax fontanarrosai* sp. n. (type series), *S*. *pinima*, and *S*. *uruguayus*. Mean ± SD (range). See Materials and methods section for the abbreviations of measurements.

Measurements	*Scinax fontanarrosai* sp. n. Type series		*Scinax pinima*	*Scinax uruguayus*	
	Males (*n* = 78)	Females (*n* = 2)	Males (*n* = 10)	Males (*n* = 84)	Females (*n* = 6)
**SVL**	21.0 ± 0.8 (19.1–23.2)	24.2 ± 0.1 (24.1–24.2)	24.4 ± 1.5 (22.1–26.5)	24.5 ± 1.2 (22.2–27.0)	25.3 ± 1.4 (23.8–27.6)
**HL**	6.2 ± 0.3 (5.6–7.0)	6.6 ± 0.6 (6.2–7.0)	7.3 ± 0.4 (6.7–7.9)	7.6 ± 0.7 (6.4–9)	8.1 ± 0.1 (8.0–8.1)
**HW**	5.9 ± 0.3 (5.3–6.7)	6.3 ± 0.5 (5.9–6.6)	7.0 ± 0.5 (6.3–7.7)	7.5 ± 0.9 (6.2–9.1)	8.2 ± 0.6 (7.8–8.6)
**IND**	1.7 ± 0.2 (1.3–2.1)	1.8 ± 0.1 (1.7–1.9)	1.8 ± 0.1 (1.7–2.0)	1.7 ± 0.2 (1.3–1.9)	1.5 ± 0.1 (1.4–1.5)
**IOD**	2.1 ± 0.1 (1.8–2.4)	2.2 ± 0.2 (2.0–2.3)	2.7 ± 0.2 (2.5–2.9)	3.1 ± 0.7 (2.5–4.7)	4.2 ± 0.1 (4.1–4.2)
**ED**	2.0 ± 0.1 (1.7–2.2)	2.2 ± 0.3 (2.0–2.4)	2.3 ± 0.1 (2.2–2.4)	2.4 ± 0.3 (2.1–3.0)	2.4 ± 0.5 (2.2–2.5)
**END**	1.3 ± 0.1 (1.1–1.7)	1.8 ± 0.2 (1.6–1.9)	1.6 ± 0.1 (1.5–1.8)	1.8 ± 0.2 (1.6–2.2)	1.9 ± 0.1 (1.8–1.9)
**TD**	1.1 ± 0.1 (0.9–1.6)	1.2 ± 0.2 (1.0–1.3)	1.1 ± 0.1 (1.1–1.2)	1.2 ± 0.2 (1.0–1.8)	1.2 ± 0.0 (1.2–1.2)
**TL**	10.1 ± 0.3 (9.5–10.9)	11.4 ± 1.0 (10.7–12.1)	11.6 ± 1.0 (10.3–13.2)	12.1 ± 0.6 (11.1–13.1)	12.0 ± 0.8 (11.4–12.5)
**FL**	9.6 ± 0.3 (8.8–10.4)	10.7 ± 1.2 (9.8–11.5)	11.3 ± 1.2 (9.5–12.9)	13.5 ± 3.4 (10.7–19.8)	17.8 ± 0.5 (17.4–18.1)
**3FD**	0.9 ± 0.1 (0.7–1.0)	0.9 ± 0.1 (0.8–0.9)	1.0 ± 0.1 (0.9–1.1)	1.0 ± 0.1 (0.8–1.1)	0.8 ± 0.1 (0.7–0.9)
**4TD**	0.9 ± 0.1 (0.8–1.1)	1.0 ± 0.1 (0.9–1.0)	1.1 ± 0.1 (1.0–1.2)	1.0 ± 0.1 (0.9–1.2)	0.8 ± 0.1 (0.7–0.9)

#### Adult skeletal morphology

**Fig 5:**
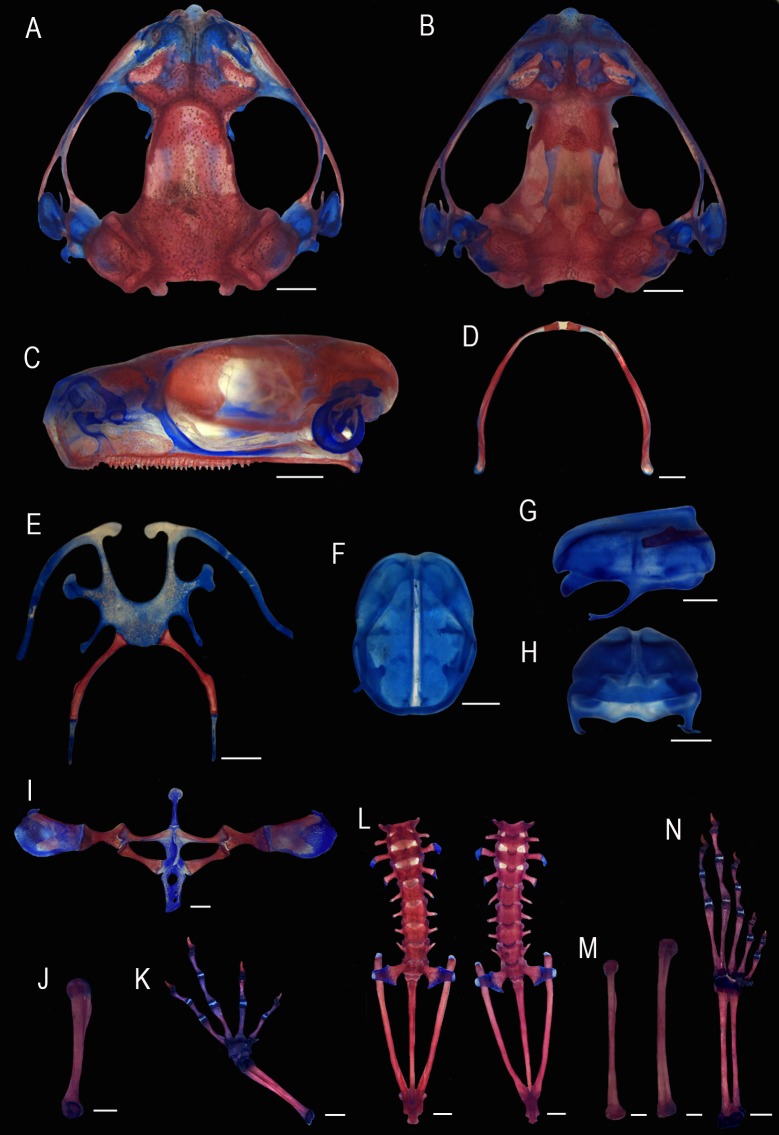
Skeletal morphology of *Scinax fontanarrosai* sp. n. (LGE 2040, male). Skull (A) dorsal, (B) ventral, and (C) lateral views. Mandible (D) dorsal view. Hyoid (E) ventral view. Larynx (F) ventral, (G) lateral, and (H) posterior views. Pectoral girdle (I) ventral view. Humerus (J) ventral view. Manus and radioulna (K) dorsal view. Vertebral column and pelvic girdle (L) dorsal and ventral views, respectively from left to right. Femur and tibiofibula, respectively from left to right (M) dorsal view. Pes (N) dorsal view. Scale bars = 1 mm.

**Fig 6:**
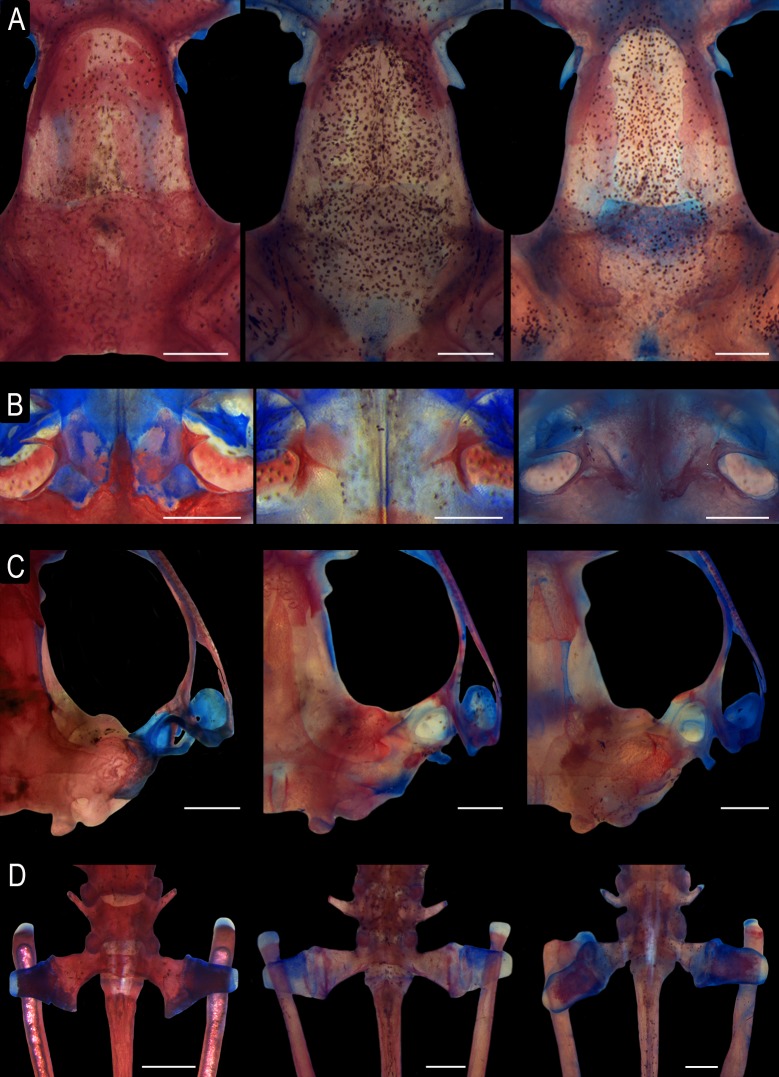
Osteological characters. (A) Dorsal view of the skull showing the level of proximity of the medial margins of the frontoparietals. (B) Shape of vomers. (C) Ventral view of the skull showing the short medial ramus of pterygoid. (D) Dorsal view of the sacral vertebra showing the medially expanded iliosacral sesamoids. *Scinax fontanarrosai* sp. n. (LGE 2040; left), *S*. *pinima* (UFMG 20185; central), and *S*. *uruguayus* (LGE 4569; right). Scale bars = 1 mm.

Descriptions based on skeletons of six adult male specimens (LGE 2040, 4486–90). No traces of skeletal hyperossification. Skull slightly wider than long (Fig 5A and 5B). Nasals L-shaped; anterior portion covering the *tectum nasi* only; inner margins at level of longitudinal axes that runs through superior prenasal cartilages; posterior margins barely overlapping the sphenethmoid posteriorly. Maxillary process terminating between postnasal wall and *planum antorbitale*, not articulating with the maxilla. Sphenethmoids dorsally smooth; anterior portion incorporating the proximal half of the *septum nasi*; laterally extending to the level of (but not including) the anterior process of the postnasal wall; and posteriorly delimiting the anterior margin of the frontoparietal fontanelle at the level of the orbitonasal foramen. *Crista supraorbitalis* short. The medial margins of the frontoparietals vary from being juxtaposed (LGE 4487) to slightly separated (LGE 2040; Figs 5A and 6A) at the level of the fontanelle; the fontanelle is almost completely concealed within this limited variation. The medial margins of frontoparietals can be irregularly indented (LGE 2040, 4486, 4488) or straight (LGE 4487–4489). *Lamina perpendicularis* poorly developed, reaching (LGE 2040) or almost reaching (LGE 4486, 4488–90) the level of the *orbitonasal foramen*. Frontoparietals not articulating medially over the *tectum synoticum*, with widely separated and roughly straight inner margins (Figs 5A and 6A). *Tectum synoticum* entirely (LGE 2040, 4487) or partially ossified (LGE 4488). Prootics narrow, medially separated by a strip of cartilage (LGE 4487–8) or fused (LGE 2040); crista parotica poorly ossified laterad and separated from the otic plate of squamosal by a narrow gap. *Crista parotica* with a cartilaginous, slender posterolateral process that extends posteriorly from its posterolateral margin.

Premaxilla anteriorly inclined; *pars palatine* shallow, barely noticeable, with a developed palatine process; *pars dentalis* with 12–17 pedicellate teeth continuously distributed along its entire extension. Alary processes narrow, rectangular shaped, anteriorly inclined, the base slightly wider than the tip, not articulating with the nasals, and with its longitudinal axes laterally directed. Maxilla weakly overlapping the lateral portion of the *pars dentalis* of the premaxilla, and posteriorly reaching (LGE 4488, 4490) or surpassing (LGE 2040, 4486, 4489) the anterior margin of the tympanic annulus. *Pars facialis* of maxilla in one plane only; two times higher than the *pars dentalis*, reaching approximately half of the distance between maxilla and *processus lingularis* of the nasal cartilage, and expanding posteriorly into a preorbital process. *Pars dentalis* with 25–34 pedicellate teeth that extend posteriorly to the anterior end of the pterygoid fossa. Maxilla overlaps approximately half of the quadratojugal length, which extends to the anterior end of the pterygoid fossa. The posterior part of the quadratojugal contacts the ventral ramus of squamosal and palatoquadrate cartilage; basal process rounded, non-bicapitate.

Squamosal composed of ventral, zygomatic, and otic rami. Ventral ramus posteriorly arched and directed posteroventrally forming an angle of 70° with the horizontal axis of the skull (Fig 5C). The distal end of the ventral ramus invests the palatoquadrate cartilage and articulates with the quadratojugal. Zygomatic ramus short, flat, pointed, directed anteroventrally, and not articulating with maxilla. Otic ramus flat, pointed in lateral view, slightly longer than the zygomatic ramus, expanded into an otic plate that does not overlap the ossified portion of *crista parotica*. Pterygoid triradiate; medial ramus short, not in contact with the otic capsule and concealing less than half of the anterior face of the basal process; posterior ramus elongate, reaching the distal portion of palatoquadrate; anterior ramus elongated, investing the maxilla laterally (Figs 5B and 6C).

Vomer with four processes: the anterior process directed towards premaxilla, separated from the maxillary arch; the prechoanal and postchoanal processes limiting the medial margin of the choanas; and the dentigerous process (Figs 5B and 6B). The latter is represented by a laminar structure without teeth (vomers edentate); it can be continuous to the main body of vomer (LGE 2040, 4488), contiguous to the main body by a medial, constricted portion (LGE 4486, 4490), or separated from the main body of vomer (LGE 4487). The medial margins of vomers are slightly convex posteriorly and widely divergent anteriorly. Palatines reduced to thin slivers. Parasphenoid with a pointed (LGE 4487–8, 4490) or rounded (LGE 2040) cultriform process, that does (LGE 2040) or does not (LGE 4487, 4490) reach anteriorly the level of the orbitonasal foramen; alary processes oriented posterolaterally, short, reaching the level of condyloid fossa laterally; posteromedial process extremely reduced, shorter than half the distance between the posterior margin of alary process and *foramen magnum*. Exoccipitals separated by a strip of cartilage. Oval occipital condyles perpendicularly oriented in relation to the main body axis, flanking the foramen magnum ventrolaterally.

Tympanic annulus incomplete dorsally, without contact with the *crista parotica*. *Pars externa plectri* elongated, mediodistally expanded; *pars media plectri* slightly expanded distally and with an ossified stapedial footplate that fills the rostral portion of the *fenestra ovalis* and contacts the operculum; a cartilaginous, round, and flat *pars interna plectri* that lies in the rostral half of the *fenestra ovalis* and overlaps the dorsal portion of the operculum.

*Septum nasi* narrow, vertical, bearing an anterior, median prenasal process that extends anteroventrally surpassing the alary process of the premaxillar: triangular (LGE 4488) or rounded (LGE 2040, 4487) in dorsal view. Alary cartilage cup-shaped, squared, and synchondrotically fused to the superior prenasal cartilage. Superior prenasal cartilage short, rectangular, and extending anteroventrally to abut the dorsomedial margin of the posterior surface of the alary process of the premaxilla. Inferior prenasal cartilage extending anteroventrally from the *solum nasi* to the posterior side of the alary process of the maxilla; it contacts the alary process on the basal portion of its posterior surface. The portion of the inferior prenasal cartilage that articulates with the *solum nasi* is oriented mediolaterally. The oblique cartilage is a semicircular cartilaginous sheet that lies lateral to each side of the *septum nasi*; it extends from the ventrolateral to the dorsomedial aspect of the nasal capsule. The ventrolateral portion of the oblique cartilage, the *planum terminale*, is a vertical plate of cartilage, with a fenestra. The inferior edge of the *planum terminale* has a caudal, rod-shaped, short, lingular process. The *crista subnasalis* is elongated, with a posteriorly opened V-shaped channel. The septomaxilla is located within the nasal capsules, and posteroventral to the corresponding alary cartilage.

Dentary lacking odontoids; angulosplenial bearing a poorly developed, laminar coronoid process in the prearticular region. Meckel’s cartilage lies between the dentary and the angulosplenial, extending posteriorly from the anterior end of the mandible, where it articulates with the mentomeckelian bone (Fig 5D). The hyoid plate is cartilaginous, slightly mineralized posteromedially in some individuals (LGE 2040, 4487, 4490), and 1.5–2 times wider than long (Fig 5E). Hyoglossal sinus U-shaped, deep, wide; the posterior margin at the level of the anterior margin of the posterolateral process. Anterior process present, laminar, slightly expanded distally, and medially curved. Anterolateral process slender, expanded distally. Posterolateral process slender, expanded distally, about three times shorter than the total length of posteromedial process. Posteromedial process abuts directly on hyoid plate (no cartilaginous stalks), and with a long cartilaginous epiphysis, one third of the posteromedial process length. Hyales slightly divergent anteriorly, and attached to the otic capsule on the skull.

Larynx oriented slightly ventral in relation to the posteromedial processes. Arytenoids oval with a slight medial constriction in dorsal view. Dorsomedial prominence of the arytenoids well developed, right triangle shaped, with major axis parallel to arytenoids (Fig 5G). Arytenoid concavity with one medial internal buttress supporting the frenulum, bounded at each end by a process for attachment of the vocal cords. Cartilaginous support rods on each distal end of the vocal cords (Fig 5F). Fibrous masses evident but not chondrified (absence of alcian blue and red). Cricoid ring with the bronchial process slender, elongated, with the distal end bifid; esophageal process absent (Fig 5H); cardiac process laminar, width similar or slightly thinner than adjacent parts of the ring, and not curved ventrally (Fig 5F). Posterior portion of the cricoid ring not cardiacly elongated.

The pectoral girdle has a typical arciferal arrangement (Fig 5I). The epicoracoids are synchondrotically fused to one another anteriorly to the interclavicle region; posteriorly to the clavicles they are free and overlapping; the right epicoracoid overlaps the ventromedial margin of the left epicoracoid and may slide over it. Omosternum and sternum cartilaginous. The former is distally expanded; the latter distally simple (LGE 2040, 4487) or bilobed (LGE 4488, 4490). Clavicle slightly longer than coracoid and scapula, with the anterior margin concave and posterior margin convex. Scapula 3/4 of clavicle length, with the anterior and posterior margins concave. *Pars acromialis* subspherical and slightly more expanded than the *pars glenoidalis*, which is concave. Anterior process of suprascapula present. Cleithrum consists of a narrow, strip of bone that encompasses the anterior margin of the suprascapula. Scapula and clavicle, as well as clavicle and coracoid separated by cartilage. Coracoids are slightly smaller than clavicles; the medial head is slightly more expanded that the lateral head; coracoid ridge present.

Humerus with a developed *crista ventralis*, the length is approximately one third of the entire arm (Fig 5J). The radio-ulna is about 75% of the length of the humerus. The manual autopodium has six individual carpal elements: Ulnare, Radiale, Element Y, Distal Carpal 3–4–5, Distal Carpal 2, and proximal element of prepollex (Morphology C of Fabrezi [32]). First distal prepollical element subcylindrical; two distal prepollical elements. Intercalary elements between ultimate and penultimate partially mineralized, forming a thick disc with concave articular surfaces facing both phalanges (Fig 5K). Distal phalanx claw-shaped, with unexpanded tip. Relative metacarpal length IV > III > V > II. Phalangeal formula 2–2–3–3.

Vertebral column with eight procoelus, nonimbrincate, presacral vertebrae (Figs 5L and 6D). Transverse process inclined anteriorly in presacral vertebrae II, III, VII, and VIII; inclined posteriorly in presacral vertebrae IV and V, and perpendicular (LGE 4487–8, 4490) or inclined posteriorly (LGE 2040, 4486) to body axis in Presacral Vertebra VI. Sacral diapophysis rounded, index maximum/minimum length of diapophysis < 3.5. Posteromedial process on the posterior margin of the sacral diapophysis absent (LGE 2040; Fig 5L), poorly developed, reduced to a small bump (LGE 4487), or developed (LGE 4488). Distal ends of the sacral diapophysis without contact with the anterior shaft of ilium; the articulation between these elements is through a partially ossified sacral sesamoid element that lies laterally to the distal terminus of each diapophysis. Sacral sesamoids are slightly expanded medially with the transversal axis at least two times longer than their longitudinal axis (Figs 5L and 6D). Pelvic girdle V-shaped in dorsal view, and composed of three pairs of elements, the ilia, ischia, and pubes, which unite in a medial symphysis (Fig 5L). Ilium about 55% of the vertebral column length; dorsally, it bears a longitudinal crest that extends for 65% of their length. A small ilial protuberance is located anterior to the level of the anterior border of the acetabulum. Ischium with a well developed and dorsally flattened interischiadic crest. Pubis markedly mineralized.

Femur length is about 26% the length of the entire limb. Tibiofibulae slightly longer than the femur (Fig 5M). The pedal autopodioum includes five prehallical elements (Tibiale, Fibulare, Element Y, Distal Tarsal 2–3, and Distal Tarsal 1) with a similar arrangement to that described by Fabrezi [33] for other hylids. Proximal prehallux ossified and distal 3–4 prehallical elements partially mineralized. The basal portions of metatarsi IV and V are in close contact. Relative metatarsal length IV > IV > III > II > I. Phalangeal formula 2–2–3–4–3. Distal phalanx claw-shaped, with an unexpanded tip. Intercalary elements between ultimate and penultimate partially mineralized (Fig 5N).

#### Tadpole description

**Fig 7:**
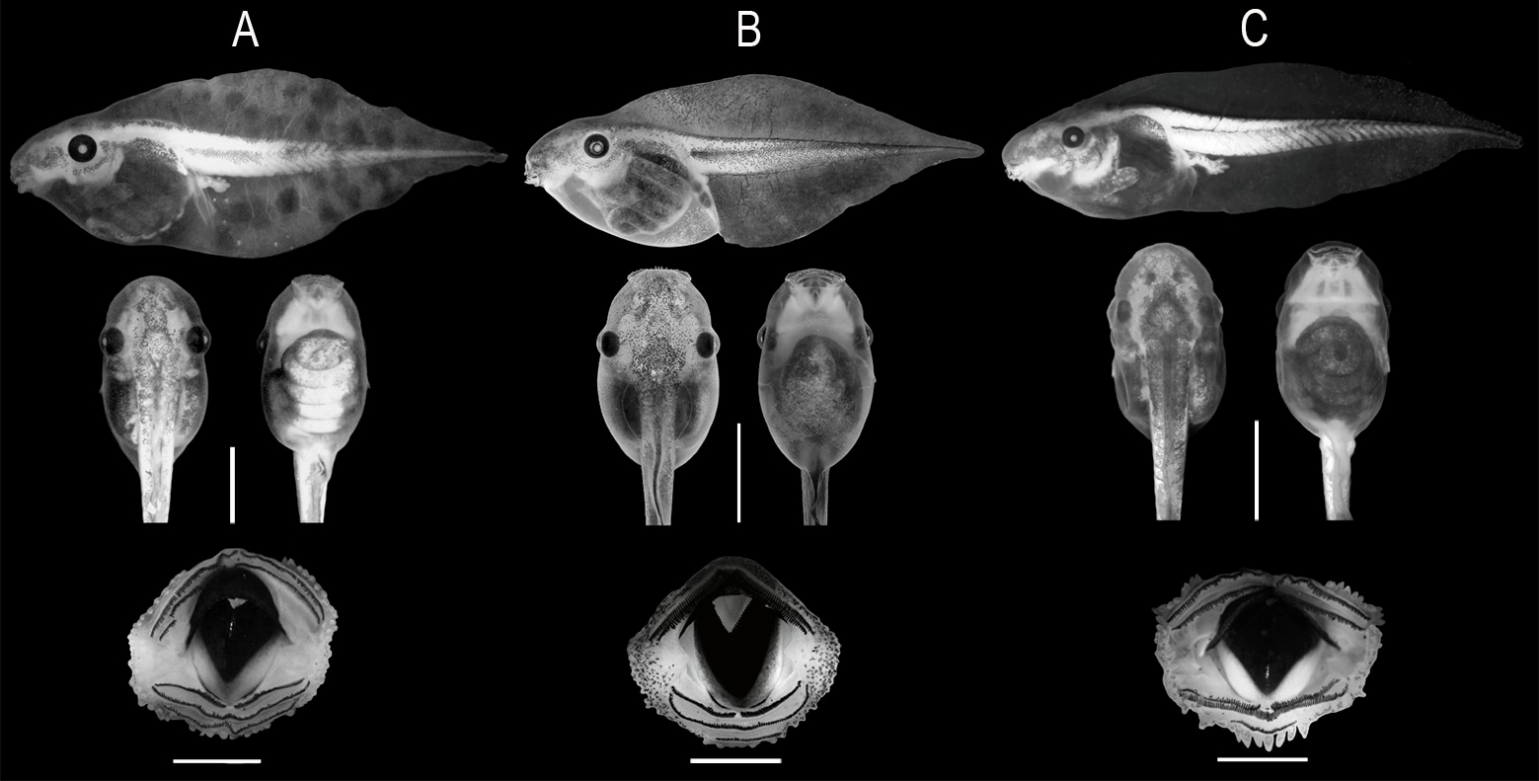
Tadpoles in different views and oral discs. (A) *Scinax fontanarrosai* sp. n. (LGE 8529, stage 31), (B) *S*. *pinima* (UFMG 2262, stage 28), and (C) *S*. *uruguayus* (MNHN 9884, stage 36). The two plates in the specimen of *S*. *uruguayus* (MNHN 9884) are covered by the folded oral disc. Scale bars = 5 mm (upper) and 1 mm (lower).

Measurements taken from 12 tadpoles at stages 35–37 (Lot LGE 8529) are presented in Table 2. Description is based on 12 tadpoles at stages 31–37 (Lot LGE 8529; Fig 7A). Body slightly higher than wide (BMH/BMW = 1.07 ± 0.09, 0.88–1.18); body length about a third of the total length (BL/TL = 0.33 ± 0.02, 0.30–0.36); body shape oval in dorsal view, with a constriction behind the eyes, and maximum width at the eye level or at the middle of the body (Fig 7A). In lateral view, ventral and dorsal contours of the body convex. Snout rounded in dorsal and sloping in lateral view. Nostrils rounded with a slightly elevated fleshy marginal rim without projections, dorsolaterally positioned (IND/BWE = 0.49 ± 0.01, 0.48–0.50), closer to the eyes than to the tip of the snout (FN/END = 1.53 ± 0.14, 1.36–1.78), placed in depressions, and visible in dorsal, lateral, and frontal views. Eye large (ED/BWE = 0.23 ± 0.01, 0.22–0.24), lateral (IOD/BWE = 0.91 ± 0.01, 0.89–0.92), and visible in ventral view. Spiracle single, lateral, sinistral, and short; inner wall fused to the body; opening oval, slightly elevated, with a diameter smaller than the tube diameter. It is placed between the middle and last third of the body (RSD/BL = 0.67 ± 0.02, 0.65–0.72), directed posterodorsally, and visible in lateral and ventral views. Intestinal coiling axis subparallel to the main body axis. Vent tube attached dextrally to the ventral fin along its entire length; it is short and does not reach the free margin of the fin. Tail long (TAL/TL = 0.67 ± 0.02, 0.64–0.70) with fins higher than body height (FH/BMH = 1.58 ± 0.12, 1.43–1.83); maximum height between first and second thirds; free margins of fins convex. Dorsal fin originating on the body, behind the eyes. Ventral fin originating from a saccular structure at the end of the abdomen. Tail axis straight, tail end acute, and tail musculature reaching the tail end.

**Table 2:** Measurements (in mm) of tadpoles. *Scinax fontanarrosai* sp. n. (LGE 8529), *S*. *pinima* (UFMG 2262, ZUEC 11536), and *S*. *uruguayus* (ZVCB 10235, 10237, and 10240). Mean ± SD (range). Measurements of *S*. *uruguayus* were taken from Kolenc et al. [6]. See Materials and methods section for the abbreviations of measurements. Abbreviation: st. = stage (s).

Measurements	*Scinax fontanarrosai* sp. n. (*n* = 12, st. 35–37)	*Scinax pinima*		*Scinax uruguayus*
		(*n* = 10, st. 28–30)	(*n* = 2, st. 37)	(*n* = 15, st. 31–33)
**TL**	29.8 ± 2.3 (26.4–32.6)	21.3 ± 1.0 (19.7–22.6)	33.6 ± 0.6 (33.2–34.1)	29.9 ± 2.1 (27–33.5)
**BL**	9.8 ± 0.3 (9.3–10.3)	7.9 ± 0.4 (7.3–8.5)	11.5 ± 0.2 (11.4–11.7)	9.5 ± 0.7 (8.5–10.9)
**BMH**	6.9 ± 0.5 (6.0–7.6)	5.9 ± 0.2 (5.6–6.4)	7.9 ± 0.4 (7.6–8.2)	6.8 ± 0.7 (5.7–7.7)
**BMW**	6.4 ± 0.4 (5.7–6.8)	5.5 ± 0.3 (5.0–6.1)	7.9 ± 0.6 (7.4–8.3)	6.4 ± 0.5 (5.6–7.2)
**TAL**	20.0 ± 2.1 (17.1–22.6)	13.4 ± 0.8 (12.1–14.4)	22.1 ± 0.39 (21.9–22.4)	20.2 ± 1.3 (18.2–22.0)
**FH**	10.8 ± 0.6 (9.6–11.9)	8.0 ± 0.5 (7.4–8.9)	12.6 ± 0.4 (12.3–12.9)	8.5 ± 0.7 (7.4–10.0)
**BWN**	4.9 ± 0.1 (4.7–5.1)	4.2 ± 0.4 (3.2–4.7)	6.1 ± 0.2 (5.9–6.2)	5.2 ± 0.2 (4.9–5.7)
**BWE**	6.4 ± 0.2 (6.0–6.7)	5.0 ± 0.3 (4.6–5.6)	7.5 ± 0.1 (7.4–7.6)	6.4 ± 0.6 (5.5–7.3)
**TMH**	2.7 ± 0.2 (2.5–3.0)	1.8 ± 0.1 (1.8–2.1)	3.1 ± 0.1 (3.0–3.2)	–
**TMW**	2.8 ± 0.3 (2.3–3.2)	1.6 ± 0.2 (1.4–1.8)	2.3 ± 0.1 (2.3–2.4)	2.8 ± 0.3 (2.3–3.2)
**ED**	1.5 ± 0.1 (1.4–1.5)	0.9 ± 0.1 (0.8–1.1)	1.1 ± 0 (1.1–1.1)	1.4 ± 0.2 (1.1–1.6)
**RSD**	6.6 ± 0.3 (6.0–6.8)	7.5 ± 0.4 (7.0–8.3)	8.4 ± 0.1 (8.3–8.5)	6.9 ± 0.4 (6.4–7.8)
**END**	1.1 ± 0.1 (0.9–1.2)	1.7 ± 0.1 (1.5–1.9)	2.1 ± 0.1 (2.0–2.2)	1.4 ± 0.2 (1.0–1.7)
**FN**	1.6 ± 0.1 (1.4–1.8)	1.4 ± 0.1 (1.3–1.6)	1.8 ± 0.2 (1.6–1.9)	1.7 ± 0.2 (1.4–2.2)
**ND**	0.3 ± 0.1 (0.2–0.3)	0.2 ± 0.0 (0.2–0.2)	0.16 ± 0.0 (0.2–0.2)	0.2 ± 0.1 (0.2–0.3)
**IOD**	5.8 ± 0.2 (5.5–6.0)	3.6 ± 0.2 (3.4–3.9)	5.1 ± 0.1 (5.0–5.1)	6.4 ± 0.6 (5.5–7.3)
**IND**	3.1 ± 0.1 (3.0–3.2)	2.4 ± 0.2 (2.1–2.8)	3.2 ± 0 (3.2–3.3)	2.8 ± 0.1 (2.6–2.9)
**ODW**	2.8 ± 0.1 (2.5–3.0)	2.5 ± 0.2 (2.2–2.7)	3.2 ± 0 (3.2–3.2)	3.3 ± 0.4 (2.9–4)
**DG**	1.1 ± 0.2 (0.7–1.4)	1.8 ± 0.1 (1.6–1.9)	1.7 ± 0.1 (1.6–1.7)	1.2 ± 0.2 (0.8–1.8)

Oral disc anteroventral, not visible dorsally, small (OD/BMW = 0.44 ± 0.02, 0.40–0.49), with one sub-angular constriction on each side (Fig 7A). Marginal papillae with a small dorsal gap (DG/ODW = 0.37 ± 0.06, 0.27–0.47), in single row on upper and lower labia and double at the angular regions. Submarginal commissural papillae present. Papillae simple, longer than wide, sub-conical; those on the posterior margin of the oral disc longer and not so tightly arranged as the others; submarginal papillae shorter and blunt. Upper jaw sheath U-shaped, lower jaw sheath with V-shaped free margin. Jaw sheaths massive, well developed, serrated, and heavily pigmented on the distal two thirds. Labial tooth row formula 2(2)/3(1). Row A1 typically bent with an angle directed to the front; P3 slightly shorter than P2; one specimen with a short ridge with labial teeth between A1 and A2. Thin, keratinized, and dark colored sheets placed between the lower jaw sheath and P1, on each side of the midline.

#### Tadpole coloration in preservative

The general appearance of tadpoles is that of scarcely pigmented anuran larvae, with distinct black spotted fins and a fine longitudinal black bands, and dark abdomen. All flecks, lines, and blotches are brown in preservative. The body is mainly translucent, with a lateral longitudinal narrow line visible from almost the tip of the snout that surrounds the eyes ventrally, and continues over the upper edge of the abdominal region and extends on the caudal myomers for about 2/3 of the tail (approximately occupying their middle third of height). Some scattered small flecks occur in the posterior half of the tail. In dorsal view, two narrow lines extend over the dorsal surface of body and tail, on both sides of the dorsal fin and reaching the tail end. Anteriorly, they are roughly continuous with a large blotch of the interocular region in correspondence with the chondrocranium. Two small semilunar blotches are present in the internarial region along with some tiny stains. In ventral view, a narrow line is evident on both sides of the ventral fin, all over the tail. The body wall is completely translucent in the ventral and most of the lateral region of the abdomen, and in the buccopharyngeal area. The oral disc is almost devoid of pigment. Although scarcely pigmented, the abdomen is dark in lateral and ventral views given the presence of some blotches in the skin of the upper and posterior portions, but mainly due to the viscera evident through the translucent body wall. The spiracle and vent tube are also unpigmented. Hind limbs are scarcely pigmented, mostly on the dorsal surface. Both fins are conspicuously colored with scattered blotches surrounded by tiny flecks that concentrate posterior to the body-tail junction, particularly over the free margins and on its last third. The anterior portion of the ventral fin concealing the vent tube is unpigmented. Both fins are transparent at their distal ends.

#### Buccopharyngeal morphology of tadpoles

Buccopharyngeal morphology and musculoskeletal system were described by Alcalde et al. [53] as *Scinax* aff. *pinima*.

#### Advertisement call

**Fig 8:**
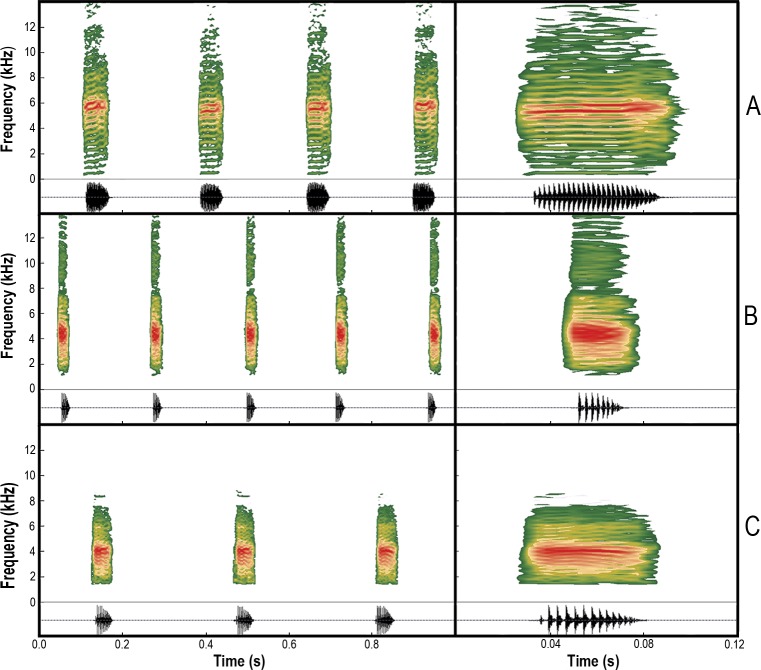
Audiospectrograms (above) and oscillograms (bellow) of the advertisement calls. (A) *Scinax fontanarrosai* sp. n. (LGE 4451), (B) *S*. *pinima* (UFMG 20184), and (C) *S*. *uruguayus* (MNHN 9877). Left: A series of calls. Right: A single call.

We analyzed calls (LGE-B 50–4) of five males: LGE 4451 (holotype), one topotype specimen (unvouchered), and three (LGE 4387 (paratype) and two unvouchered specimens) from Ruta Nacional 12, 2.6 km NE San Borjita (27.477167° S, 56.073861° W; 142 m a.s.l.), Departamento Ituzaingó, Corrientes, Argentina. Air temperature was 21.7–23.1°C. The advertisement call consists of a single, pulsed, and very highly pitched note, emitted at a rate of 3.9–4.9 notes/s (Fig 8A; Table 3). The note duration is 49–66 ms, with intervals between notes of 130–241 ms. Each note is composed of 25–31 pulses that are increasingly modulated for the first quarter, remaining with a relatively constant amplitude in the second quarter and then start decreasing up to the end of the note. Pulses are released at a rate of 490–540 pulses/s. The notes have harmonic structure (fundamental frequency 432–582 Hz). The dominant frequency is 5513–6159 Hz.

**Table 3:** Advertisement calls.

Characteristics	*Scinax fontanarrosai* sp. n.	*Scinax pinima*			*Scinax uruguayus*		
	Argentina (*n* = 5)	Brazil: SC (*n* = 1)	Brazil: MG (*n* = 1)	Brazil: MG (*n* = 6)	Brazil: RS (*n* = 1)	Uruguay (*n* = 13)	Uruguay (*n* = 1)
**Number of calls**	261	50	–	284	50	653	–
**Dominant frequency (Hz).**	5774 ± 162 (5513–6159)	4342 ± 50 (4242–4457)	3300	4160 ± 166 (3919–4479)	4138 (3858–4332)	4201 ± 209 (3833–4651)	4146 ± 128 (3805–4329)
**Note duration (ms)**	39 ± 3 (49–66)	40 ± 0 39–43	~100	39 ± 3 (30–43)	19.42 (16–23)	23 ± 0 (17–28)	22 ± 2
**Note rate (s)**	4.3 ± 0.4 (3.9–4.9)	3.32	–	3.21 ± 0.1 (3.0–3.3)	–	4.3 ± 0.4 (3.7–5.0)	–
**Interval note (ms)**	179 ± 24 (130–241)	266 ± 20 (238–326)	~200	280 ± 20 (260–330)	202 (132–256)	216 ± 27 (157–290)	–
**Pulse number**	27 ± 2 (25–31)	12 ± 1 (11–13)	–	12 ± 1 (11–14)	7.24 (6–9)	12 ± 1 (7–10)	8* (7–10)
**Pulse rate (ms)**	510 ± 15 (490–540)	282 ± 10 (275–310)	–	326 ± 13 (308–355)	373 (272–391)	378 ± 46 (286–476)	–
**References**	Present work	Present work	1	Present work	2	Present work	3

Mean ± SD (range). References: 1 = Bokermann and Sazima [5]; 2 = Kwet [17]; and 3 = Kolenc et al. [6]. Abbreviations: MG = Minas Gerais, SC = Santa Catarina, and RS = Rio Grande do Sul.

* = Mode.

#### Cytogenetics

**Fig 9:**
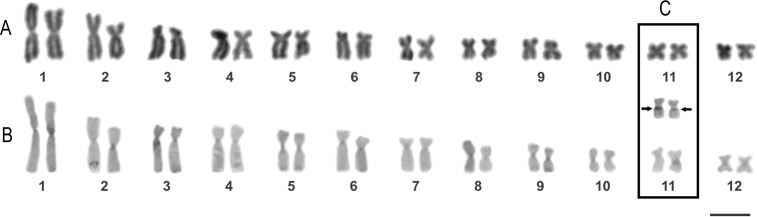
Chromosome morphology of *Scinax fontanarrosai* sp. n. (LGE 4472). (A) Conventional staining. (B) C-banding technique. (C) Ag-NORs. Arrows indicate the NOR bearing chromosome pair. Scale bar = 10 μm.

Descriptions are based on six adult males (LGE 4385–6, 4471–3, 4489). *Scinax fontanarrosai* sp. n. has a diploid karyotype composed of 12 biarmed chromosome pairs (2*n* = 2*x* = 24; FN = 48), without evident heteromorphic sex chromosomes (Fig 9). The pairs 1, 2, 8, 9, 10, and 12 are metacentric and the others submetacentric (Fig 9A; Table 4). The SCs are located on the proximal region of long arms of Pair 11 and the sparse c-positive bands are distributed exclusively on the centromeres in all chromosome complement (Fig 9B). The NORs are coincident with secondary constrictions, over the long arms of Pair 11 (Fig 9C).

**Table 4:** Morphometric analysis of chromosomes. *Scinax fontanarrosai* sp. n. (above) and *S*. *uruguayus* (below; data taken from Cardozo et al. [103]. Abbreviations: AR = arm relation, CI = centromeric index, SD = standard deviation, CT = chromosome, type: *m* = metacentric, and *Sm* = submetacentric.

**Chromosome pair of *Scinax fontanarrosai* sp. n.**												
	**1**	**2**	**3**	**4**	**5**	**6**	**7**	**8**	**9**	**10**	**11**	**12**
**% Set**	14.00	12.70	10.91	10.04	9.70	7.88	6.32	6.30	5.81	5.43	5.60	5.32
**AR ± SD**	1.16±0.08	1.60±0.10	1.99±0.07	2.57±0.18	2.19±0.10	2.55±0.17	1.94±0.08	1.14±0.10	1.24±0.12	1.26±0.18	1.90±0.17	1.15±0.13
**CI ± SD**	0.46±0.02	0.39±0.01	0.33±0.01	0.28±0.01	0.31±0.01	0.28±0.01	0.34±0.01	0.47±0.02	0.45±0.03	0.45±0.04	0.35±0.02	0.47±0.03
**CT**	***m***	***m***	***Sm***	***Sm***	***Sm***	***Sm***	***Sm***	***m***	***m***	***m***	***Sm***	***m***
**Chromosome pair of *Scinax uruguayus***												
	**1**	**2**	**3**	**4**	**5**	**6**	**7**	**8**	**9**	**10**	**11**	**12**
**% Set**	14.21	12.60	10.14	9.88	9.72	7.90	6.43	6.35	5.95	5.75	5.63	5.45
**AR ± SD**	1.11±0.07	1.54±0.11	1.89±0.07	2.49±0.12	2.19±0.10	2.49±0.23	1.97±0.12	1.17±0.12	1.28±0.14	1.26±0.18	1.90±0.17	1.15±0.13
**CI ± SD**	0.46±0.01	0.41±0.01	0.35±0.01	0.29±0.01	0.31±0.01	0.29±0.02	0.34±0.02	0.46±0.02	0.46±0.04	0.45±0.04	0.35±0.02	0.47±0.03
**CT**	***M***	***m***	***Sm***	***Sm***	***Sm***	***Sm***	***Sm***	***m***	***m***	***m***	***Sm***	***m***

#### Geographic distribution

**Fig 10:**
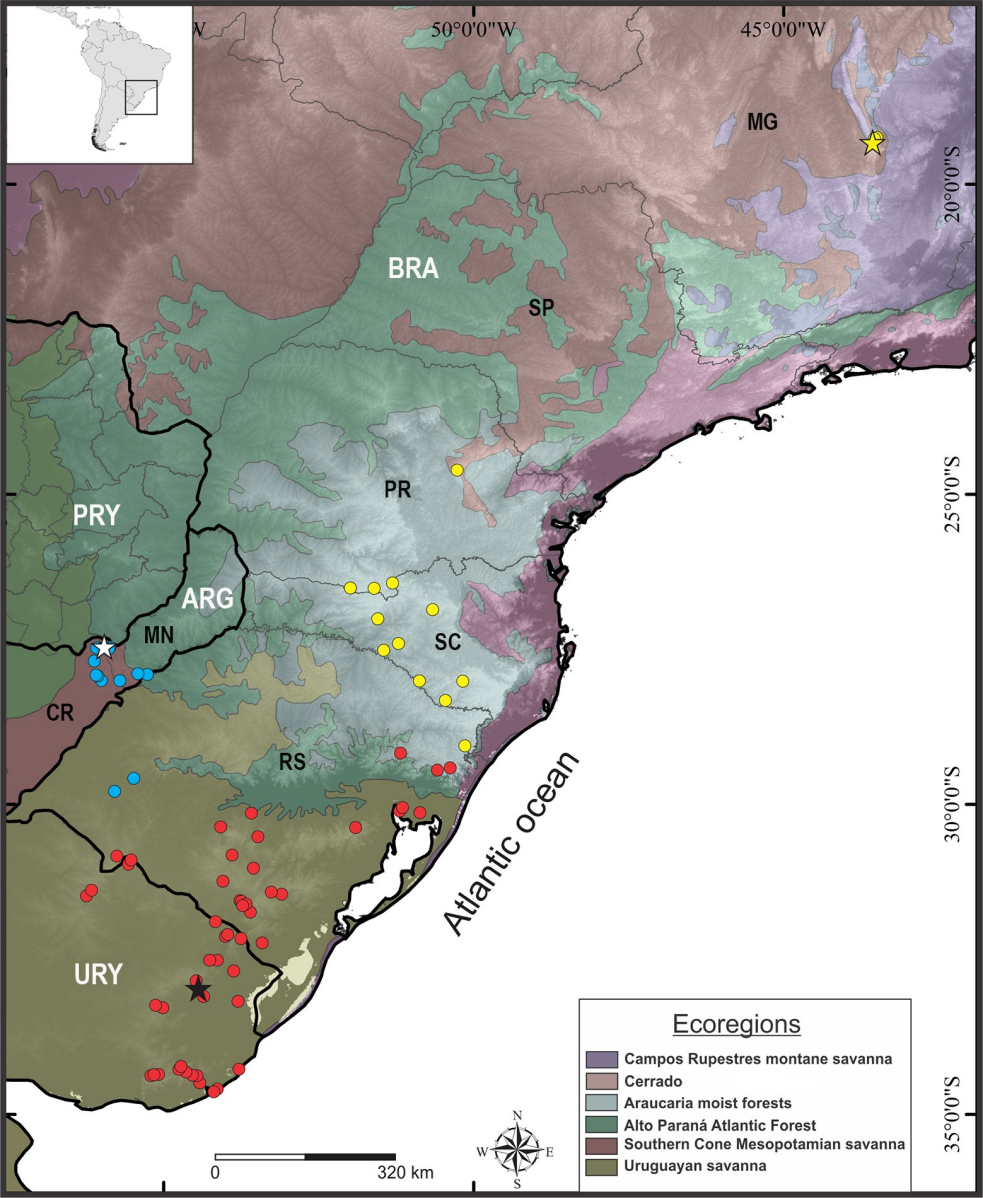
Partial map of the South American Ecoregions showing the known geographical distribution of the species of the *Scinax uruguayus* group. *Scinax fontanarrosai* sp. n. (light blue circles; white star: type locality), *S*. *pinima* (yellow circles; yellow star: type locality), and *S*. *uruguayus* (red circles; black star: type locality). Abbreviations: ARG = Argentina, BRA = Brazil, PRY = Paraguay, and URY = Uruguay. Brazilian states: MG = Minas gerais, PR = Paraná, RS = Rio Grande do Sul, SC = Santa Catarina, and SP = São Paulo. Argentinian provinces: CR = Corrientes and MN = Misiones.

*Scinax fontanarrosai* sp. n. occurs in open areas of at least ten localities in the Provinces of Misiones and Corrientes, northeastern Argentina, and two localities in the State of Rio Grande do Sul in southern Brazil (95–163 m a.s.l.). These areas are part of the Southern Cone Mesopotamian Savanna and the western part of the Uruguayan Savanna Ecoregions, respectively (sensu [58]; Fig 10).

#### Natural history

**Fig 11:**
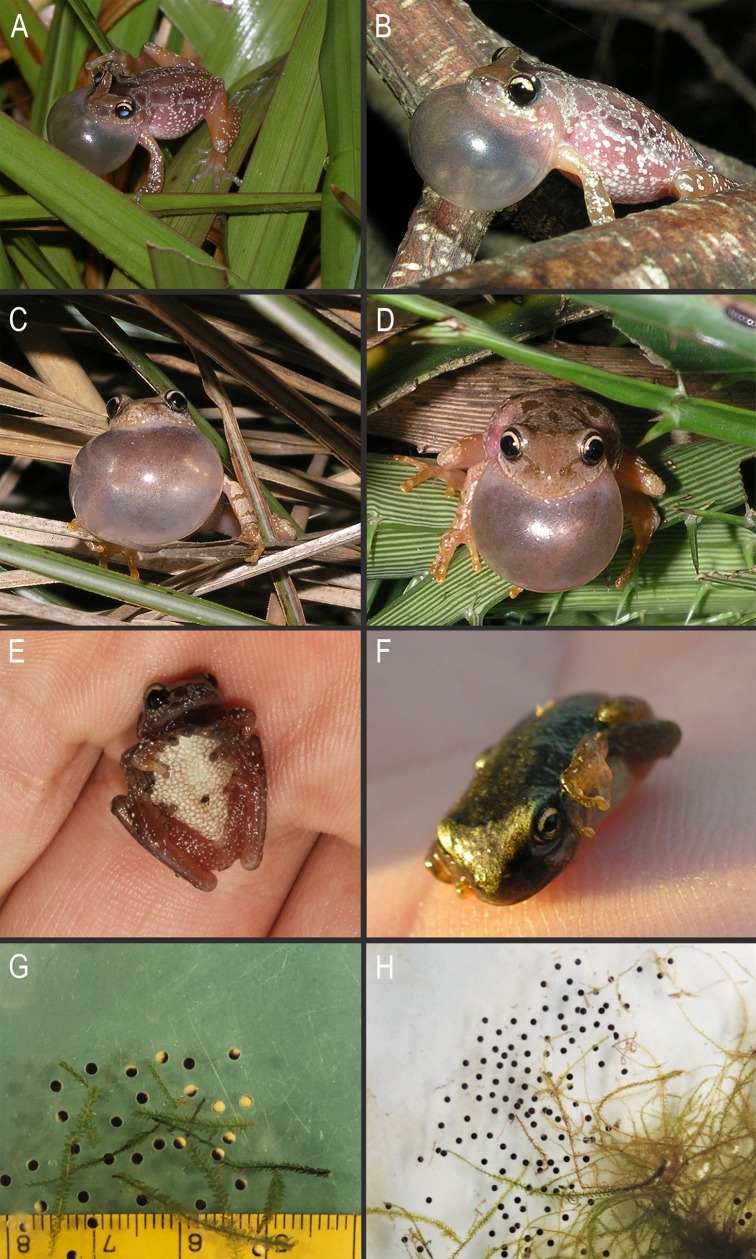
Calling males, defensive behavior, and egg clutches. Males of (A–B) *Scinax fontanarrosai* sp. n. and (C–D) *S*. *uruguayus* calling at breeding sites. Male of (E) *S*. *fontanarrosai* sp. n. and a juvenile of (F) *S*. *uruguayus* performing the passive defensive “crouching down” behavior. (G) and (H) egg clutches of *S*. *uruguayus*. Note the dark brown to black animal pole and a dark cream vegetal pole in G.

Adult specimens of *Scinax fontanarrosai* sp. n. were collected at night, when breeding in temporary ponds after heavy rains. Males called from herbaceous and shrubby vegetation perched between 5 and 120 cm high (Fig 11A and 11B). This species has explosive reproduction (sensu [59]), most commonly during spring and summer seasons (between October and early April), and was occasionally detected in reproductive activity during July. Tadpoles and juveniles were collected at the same temporary ponds where adult males are calling. Tadpoles can be included in the nektonic guild [36]. Two specimens (male and juvenile) of *S*. *fontanarrosai* sp. n. were observed performing the passive defensive behavior “crouching down” ([60]; Fig 11E).

At the type locality, *Scinax fontanarrosai* sp. n. is sympatric with *Melanophryniscus atroluteus* (Miranda-Ribeiro, 1920), *Rhinella azarai* (Gallardo, 1965) (Bufonidae), *Dendropsophus nanus* (Boulenger, 1889), *S*. *fuscovarius* (Lutz, 1925), *S*. *similis* (Cochran, 1952), *S*. *squalirostris* (Lutz, 1925) (Hylidae), *Leptodactylus fuscus* (Schneider, 1799), *L*. *gracilis* (Duméril and Bibron, 1840), *L*. *mystacinus* (Burmeister, 1861), *Physalaemus albonotatus* (Steindachner, 1864), *P*. *cuvieri* Fitzinger, 1826, *Pseudopaludicola falcipes* (Hensel, 1867) (Leptodactylidae), *Elachistocleis bicolor* (Guérin-Méneville, 1838) (Microhylidae), and *Odontophrynus* sp. aff. *americanus* (Odontophrynidae).

#### *Scinax pinima* (Bokermann and Sazima, 1973)

*Hyla pinima*.—Bokermann and Sazima [5], species description. Caramaschi [61]. Garcia et al. [62]. Kolenc et al. [6]. Eterovick and Sazima [13]. Faivovich et al. [3]. Giraudo et al. [14]. Lema and Martins [63].

*Hyla uruguaya*.—*non* Schmidt [15]. Langone [11], *partim*. Kwet and Di-Bernardo [64], *partim*. Bernarde [18], *partim*. Eterovick and Sazima [13], *partim*. Giraudo et al. [14], *partim*.

*Scinax pinima*.—Faivovich et al. [3]. Leite et al. [51]. Silva et al. [65]. Alcalde et al. [53].

*Scinax uruguayus*.—Faivovich et al. [3], *partim*. Kwet et al. [52], *partim*. Marin da Fonte et al. [56], *partim*. Bolzan et al. [66]. Nogueira et al. [67].

*Julianus uruguayus*.—Duellman et al. [9], *partim*. Ferrão et al. [57], *partim*. Ferrão et al. [68], *partim*. Ferrão et al. [69].

*Julianus pinimus*.—Duellman et al. [9]. Ferrão et al. [57].

#### Holotype

MZUSP 73668 (ex WCAB 46238), adult male, by original designation (Fig 1B). Type locality: “km 132, da Serra do Cipó, Jaboticatubas, Minas Gerais, Brasil”. The type locality is currently known as Alto Palácio, Municipality of Santana do Riacho, the region previously belonging to the Municipality of Jaboticatubas as described by Bokermann and Sazima [5].

#### Paratypes examined

MZUSP 73859–63 (ex WCAB 47439–40, 47442–3, 47445), adult males from the type locality. CFBH 6241–2 (ex WCAB 47429–30), adult males from Estrada de Vespasiano a Conceição do Mato Dentro km 110–132, Minas Gerais, Brazil.

#### Additional adult specimens examined

Thirty-four males from different localities in the Brazilian states of Minas Gerais, Paraná, Rio Grande do Sul, and Santa Catarina. See S1 Appendix for a complete list of specimens examined.

#### Diagnosis

*Scinax pinima* can be differentiated from *S*. *fontanarrosai* sp. n. and *S*. *uruguayus* by the combination of (1) large size in females (SVL 29.0 mm in the only female available; [5]); (2) broadly rounded head in dorsal view (Fig 3A); (3) presence of two or three well or poorly marked interorbital grooves (Fig 3A); (4) anterior portion of the choanae not concealed by the palatal shelf of the maxillary arch when roof of mouth is viewed from below; (5) V-shaped cephalic blotch; (6) bicolored iris, with a golden upper half and a dark brown lower half, with small round, scattered, and golden chromatophores (Fig 3B); (7) bicolored discs of fingers and toes with a gray proximal half and a light brown to orange distal half in life (Fig 3C); (8) hidden surfaces of thighs and tibia light purple in life (Fig 3D); (9) frontoparietals as slender strips at the level of a completely exposed fontanelle; (10) pointed dentigerous process of the vomers without teeth; (11) palatines reduced to thin slivers (palatines overlapping about 3/4 of planum antorbitale in *S*. *uruguayus*); (12) intercalary elements between ultimate and penultimate phalanges partially mineralized; (13) larynx with oval arytenoids, without medial constriction in dorsal view; (14) advertisement call composed by a single, short (30–43 ms), and pulsed note (11–14 pulses/note), emitted at a rate of 3.0–3.3 notes/s; (15) pulse rate of 275–355 pulses/s; (16) notes with the first pulse distinctly lower than the second, and decreasing amplitude modulation from the second pulse to the last one; (17) notes lacking harmonic structure; and (18) dominant frequency between 3919–4479 Hz.

#### Comparison with *Scinax uruguayus*

*Scinax pinima* differs from *S*. *uruguayus* (character states in parentheses) by the following character states: large size in females (SVL 23.8–27.6 mm in females; *n* = 6); two or three well or poorly marked interorbital grooves (interorbital grooves absent; Fig 3A); anterior portion of the choanae not concealed by the palatal shelf of the maxillary arch (choanae concealed by the palatal shelf of the maxillary arch when roof of mouth is viewed from below); V-shaped cephalic blotch (subtriangular cephalic blotch); bicolored iris with a golden upper half and a dark brown lower half with scattered, small, round, and golden spots (iridescent golden upper half and a golden lower half with brown reticulations; Fig 3B); and bicolored discs with a gray proximal half and a light brown to orange distal half in life (discs of fingers and toes golden yellow to orange; Fig 3C).

*Scinax pinima* also differs from *S*. *uruguayus* by having a pointed dentigerous process of the vomers without teeth (thick, rectangular dentigerous process with teeth), and intercalary elements between ultimate and penultimate partially mineralized (completely mineralized). Furthermore, the advertisement call of *S*. *pinima* differs from that of *S*. *uruguayus* in the following character states: note duration ranging from 30–43 ms (17–28 ms); notes composed of 11–14 pulses (7–10 pulses), released at the rate of 3.0–3.3 notes/s (3.7–5.0 notes/s); and notes with the first pulse distinctly lower than the second, and decreasing amplitude modulation from the second pulse to the last one (notes with pulses of decreasing amplitude modulation from the first to the last one).

#### Description of holotype

See Bokermann and Sazima [5] for a description of the holotype.

#### Coloration in life of the holotype

See Bokermann and Sazima [5] for a description of the coloration in life of the holotype.

#### Coloration in preservative of the holotype

The holotype of *Scinax pinima* is well preserved. Its dorsum is dark brown colored with a light brown reticulated pattern. A brownish V-shaped cephalic blotch and dark brown colored vocal sac are visible.

#### Variation and morphological observations on the adult specimens examined

**Fig 12:**
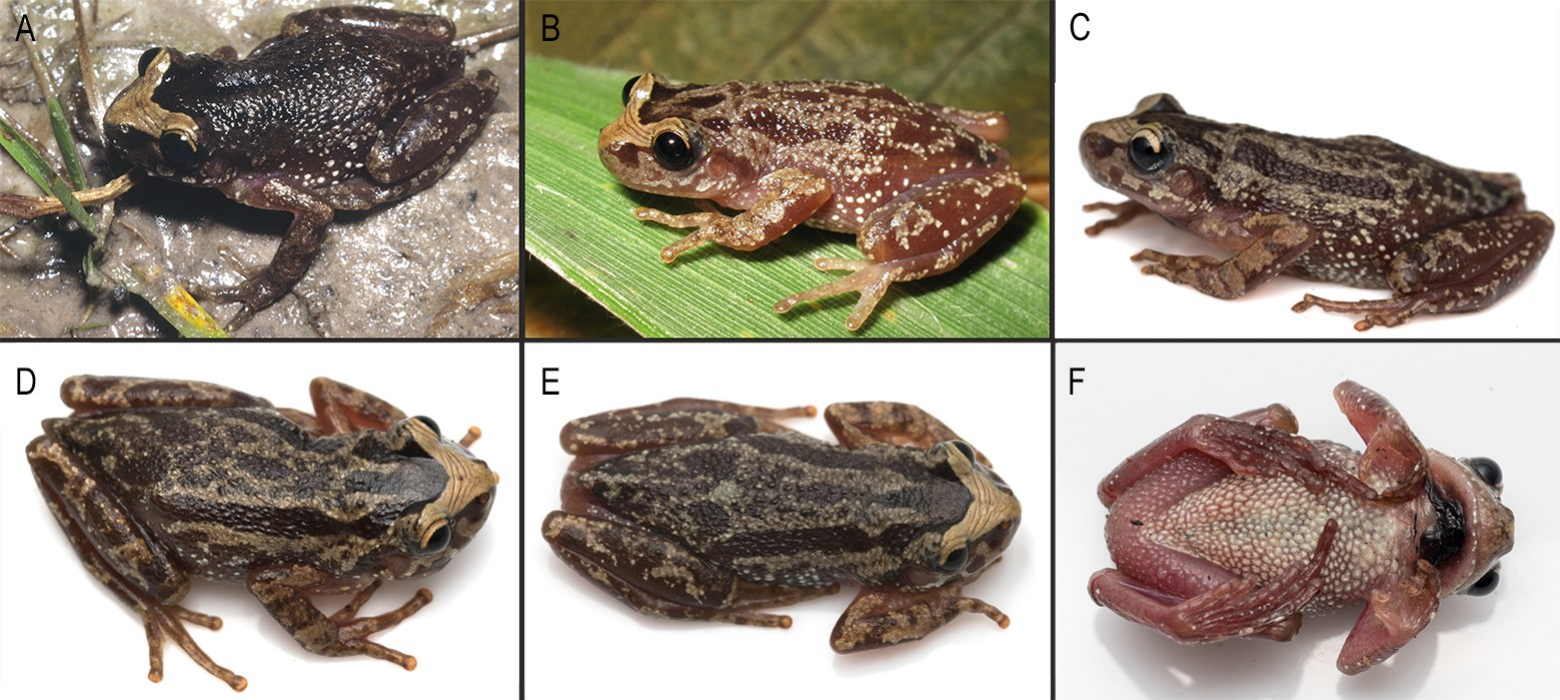
Color pattern of *Scinax pinima* in life. (A–C) Dorsolateral, (D–E) dorsal, and (F) ventral views. Photo (A) courtesy of J Pombal Jr.

Measurements of some adult specimens are presented in Table 1. We observed variation in the development of the interorbital grooves and in the degree of pigment concentration on the dorsum (Fig 12). All specimens from Serra do Cipó, Minas Gerais, Brazil have two or three well marked dermal interorbital grooves; specimens from southern populations (states of Paraná, Santa Catarina, and Rio Grande do Sul) have poorly marked grooves. Also, the degree of dorsal dark pigmentation is quite variable with some specimens lighter than others.

Males have expanded, dark brown colored vocal sacs with longitudinal folds evident in deflated position (Fig 12F), and light-colored nuptial pads without macroscopically evident epidermal projections. Pectoral fold with only a preaxillar element. The discs in hand and toes are rounded. Hand webbing is basal. Toe webbing formula I 2^+^–2^+^ II 2^-^–3^+^ III 3^+^–2^-^ IV 3^+^–2^-^ V. The only available female (SVL 29.0 mm; [5]) is larger than males (SVL 22.1–26.5 mm).

#### Adult skeletal morphology

The skeletal morphology of the only studied specimen of *Scinax pinima* (UFMG 20185) differs in some characteristics from those of *S*. *fontanarrosai* sp. n. (LGE 2040, 4486–90) and *S*. *uruguayus* (LGE 4569, 4578–81). They are as follows (character states of the other species in parentheses). (1) Nasals narrow (nasals L-shaped), not overlapping the sphenethmoid posteriorly (nasal barely overlapping the sphenethmoid posteriorly). (2) Sphenethmoid reduced; anterior portion incorporates only the base of the *septum nasi*, not laterally extended, and posteriorly covering slightly the anterior margin of the frontoparietal fontanelle (anterior portion incorporating at least half of the *septum nasi* length; laterally extending to the level of the anterior process of the postnasal wall; and posteriorly covering the anterior margin of the frontoparietal fontanelle, at the level of the orbitonasal foramen). (3) Frontoparietals as slender strips at the level of the fontanelle resulting in an almost completely exposed fontanelle (inner margins of the frontoparietals juxtaposed or slightly separated resulting in an almost completely concealed fontanelle in *S*. *fontanarrosai* sp. n.; Fig 6A). (4) *Tectum synoticum* entirely cartilaginous (*tectum synoticum* entirely or partially ossified). (5) Prootics with a non-ossified crista parotica laterad (crista parotica poorly ossified laterad). (6) Maxilla posteriorly overlapping approximately one third of the quadratojugal length (overlapping approximately half of the quadratojugal length). (7) Anterior process of the vomers absent, and prechoanal and postchoanal processes poorly developed, embracing a small portion of the medial margin of the choanas (anterior process present; prechoanal and postchoanal processes limiting the medial margin of the choanas). (8) Dentigerous process of the vomers pointed without teeth (laminar, without teeth in *S*. *fontanarrosai* sp. n. and thick, rectangular, with teeth in *S*. *uruguayus*; Fig 6B). (9) Palatines reduced to thin slivers (palatines overlapping about 3/4 of planum antorbitale in *S*. *uruguayus*). (10) Posteromedial process of the parasphenoid absent (present, but extremely reduced in *S*. *fontanarrosai* sp. n.). (11) Arytenoids oval, without medial constriction in dorsal view (arytenoids oval, with a slight medial constriction in dorsal view in *S*. *fontanarrosai* sp. n.). (12) Intercalary elements between ultimate and penultimate partially mineralized (completely mineralized in *S*. *uruguayus*).

Additionally, we observed some characteristics in the only available skeleton of *Scinax pinima* that vary intraspecifically in *S*. *fontanarrosai* sp. n. and/or *S*. *uruguayus* (character states in parentheses). These are as follows. (1) Prootics separated by a strip of cartilage medially (prootics medially fused in some specimens of *S*. *fontanarrosai* sp. n.). (2) *Pars dentalis* of premaxilla and maxilla with 12–13 and 30–32 pedicellate teeth, respectively (8–17 and 25–36 pedicellate teeth in the premaxilla and maxilla, respectively). (3) Maxilla posteriorly surpassing the anterior margin of the tympanum (maxilla posteriorly reaching the anterior margin of the tympanum in some specimens of *S*. *fontanarrosai* sp. n.), and contiguous to the main body of the vomer by a medial, constricted portion (continuous to the main body of vomer in some specimens of *S*. *fontanarrosai* sp. n. and *S*. *uruguayus*; and separated from the main body of vomer in some specimens of *S*. *fontanarrosai* sp. n.; Fig 6B). (4) Parasphenoid with a pointed cultriform process, whose anterior end is posterior to the orbitonasal foramen (rounded cultriform process with the anterior end extending to the level of the orbitonasal foramen in some specimens of *S*. *fontanarrosai* sp. n.). (5) Median prenasal process of the *septum nasi* at the level of the alary process of the premaxilla anteriorly (surpassing the alary process of the premaxillar in *S*. *fontanarrosai* sp. n. and some specimens of *S*. *uruguayus*), and rounded shaped in dorsal view (triangular in some specimens *S*. *fontanarrosai* sp. n.). (6) Hyoid plate slightly mineralized posteromedially, and two times wider than longer (entirely cartilaginous and one and a half times wider than longer in some specimens of *S*. *fontanarrosai* sp. n.). (7) Sternum distally simple (bilobed in some specimens of *S*. *fontanarrosai* sp. n. and *S*. *uruguayus*). (8) Transverse process perpendicular to body axis in the Presacral Vertebra VI (inclined posteriorly in *S*. *uruguayus* and in some specimens of *S*. *fontanarrosai* sp. n.). (9) Posteromedial process on the posterior margin of the sacral diapophysis absent (absent or poorly developed in some specimens of *S*. *fontanarrosai* sp. n. and *S*. *uruguayus*; and developed in some specimens of *S*. *fontanarrosai* sp. n.).

#### Tadpoles

Larvae of *Scinax pinima* were described by Bokermann and Sazima [5] based on specimens at stages 40–42. Subsequently, Kolenc et al. [6] based on J Faivovich´s personal observations (*in litt*.) reported valuable information on the tadpoles studied by Bokermann and Sazima [5]. Our own observations on 12 tadpoles (stages 28–30 and 37; Lots UFMG 2262 and ZUEC 11536) agree with those made by these authors (Fig 7B). Measurements from ten tadpoles at stages 28–30 (Lot UFMG 2262; Fig 7B) and two tadpoles at stage 37 (Lot ZUEC 11536) are presented in Table 2.

#### Buccopharyngeal morphology of tadpoles

The buccopharyngeal cavity has keratinized, colored, and multicuspidate spurs, behind the posterior edge of the lower jaw sheath. Multifid papillae project from the medial region of the cartilago meckeli, overlapping at the midline. The lingual anlage presents no papillae, and prepocket papillation is absent. Buccal pockets are narrow, transverse, and medially curved slits. The buccal floor arena is delimited by 3–5 papillae. The ventral velum is sinuous, with conspicuous ondulations on each side and a noticeable median notch. The glottis is exposed. The buccal roof presents prenarial arena without pustules, and choanae-like oblique slits in the posterior margin form narial valves. Postnarial arena has two long papillae and any few pustules, and a high and triangular median ridge with a pustulate margin. Lateral ridge papillae are wide, flat and pustulate. The buccal roof arena is not clearly defined, presenting numerous (~20) and scattered small pustules, and a few small lateral papillae. The dorsal velum is short, smooth, and with large secretory pits.

#### Advertisement call

We analyzed calls (CBUFMG 991, 993–7) of six topotype specimens (UFMG 20179, 20181–5). Additionally, we analyzed calls (LGE-B 69) of one specimen (LGE 21256) from Fazenda Serra da Esperança, Lebón Regis, Santa Catarina, Brazil (Table 3).

The advertisement call of *Scinax pinima* was first reported by Bokermann and Sazima [5]. Our analyses on calls (CBUFMG 991, 993–7) of six topotype specimens showed that it is composed of a single pulsed note, emitted at a rate of 3.0–3.3 notes/s (Fig 8B; Table 3). Note duration ranges from 30–43 ms, and intervals between notes from 260–330 ms. Each note is composed of 11–14 pulses that are decreasingly modulated within each one, except for the first pulse, which is consistently lower than the second. Pulses rate of 308–355 pulses/s. The notes lack harmonic structure. Dominant frequency is 3919–4479 Hz. Spectral and temporal parameters of the advertisement call of the single specimen from the State of Santa Catarina are very similar to those of topotype specimens (see Table 3).

#### Cytogenetics

No samples were available for this study.

#### Geographic distribution

*Scinax pinima* shows an interesting disjunct geographic distribution (Fig 10). The northernmost known population inhabits the mountain system of Serra do Cipó, Minas Gerais, Brazil, which belongs to the Campos Rupestres Montane Savanna Ecoregion (1100–1400 m a.s.l.). Other populations are known nearly 900 km southwards, in altitude grasslands of the Araucaria Moist Forest Ecoregion of the Brazilian states of Paraná, Santa Catarina, and northern of Rio Grande do Sul (787–1027 m a.s.l.).

#### Natural history

Some notes on the natural history of *Scinax pinima*, including comments on breeding sites, clutch structure and egg size, were provided by Bokermann and Sazima [5]. *Scinax pinima* is an explosive breeder (sensu [59]), detectable mainly after the heavy rains of the rainy season. A female kept in captivity laid a clutch with ~300 eggs (single eggs sensu [70]) in water, attached to the glass at the bottom of the aquarium [5]). The ovum has a diameter of 1.5 mm, and 1.7 mm when the viteline membrane is considered, and the egg jelly diameter is 4.5 mm [5].

The type locality of *Scinax pinima* belongs to a singular region of the Serra do Cipó, a plateau in which some shallow temporary ponds are formed during the rainy season (November to February). These ponds are used as a breeding site by *S*. *pinima* in syntopy with *Dendropsophus elegans* (Wied-Neuwied, 1824), *D*. *minutus* (Peters, 1872), *D*. *seniculus* (Cope, 1868), *Scinax curicica* Pugliese, Pombal, and Sazima, 2004, *S*. *fuscovarius*, *S*. *squalirostris*, *S*. *“x-signatus”* (Hylidae), *Leptodactylus furnarius* Sazima and Bokermann, 1978, *L*. *jolyi* Sazima and Bokermann, 1978, *L*. *latrans* (Steffen, 1815), *Physalaemus cuvieri*, *P*. *evangelistai* Bokermann, 1967, *Pseudopaludicola mineira* Lobo, 1994 (Leptodactylidae), *Elachistocleis cesarii* (Miranda-Ribeiro, 1920) (Microhylidae), *Odontophrynus juquinha* Rocha, Sena, Pezzuti, Leite, Svartman, Rosset, Baldo, and Garcia, 2017, and *Proceratophrys cururu* Eterovick and Sazima, 1998 (Odontophrynidae).

#### *Scinax uruguayus* (Schmidt, 1944)

*Hyla uruguaya*.—Schmidt [15], species description. Barrio [71]. Langguth [72]. Gudynas [73]. Achaval [74]. Langone [16]. Klappenbach and Langone [75]. Prigioni and Achaval [76]. Langone [77]. Achaval and Olmos [78]. Langone [11], *partim*. Olmos et al. [79]. Achaval [80]. Bernarde [18], *partim*. Maneyro and Langone [81]. Probides [82]. Borteiro et al. [83]. Garcia et al. [62]. Maneyro and Langone [84]. Achaval and Olmos [12]. Langone [85]. Kolenc et al. [6], *partim*. Eterovick and Sazima [13], *partim*. Langone et al. [86]. Núñez et al. [87]. Faivovich et al. [3]. Giraudo et al. [14], *partim*.

*Hyla minuta*.—*non* Peters [88]. Barrio [71], *partim*. Lutz [89], *partim*. Braun and Braun [90], *partim*. Gorham [91], *partim*. Duellman [92], *partim*. Cei [93], *partim*. Gallardo [94], *partim*.

*Hyla* sp.—Braun and Braun [95].

*Hyla pinima*.—*nec* Bokermann and Sazima [5]. Braun and Braun [96], *partim*.

*Hyla minuta uruguaya*.—Klappenbach [97].

*Scinax uruguayus*.—Faivovich et al. [3]. Achaval and Olmos [98]. Kolenc et al. [99]. Leite et al. [51], *partim*. Ziegler and Maneyro [100]. Canavero et al. [101]. Canavero et al. [102]. Kwet et al. [52], *partim*. Alcalde et al. [53]. Cardozo et al. [103]. Langone [104]. Lema and Martins [63], *partim*. Prigioni et al. [105]. Maneyro and Carreira [106]. Kolenc et al. [99]. Marin da Fonte et al. [56], *partim*.

*Julianus uruguayus*.—Duellman et al. [9], *partim*. Ferrão et al. [57], *partim*. Frost [1], *partim*.

#### Holotype

FMNH 10567, adult male, by original designation (Fig 1C). Type locality: “Quebrada de los Cuervos, Department of Treinta y Tres, Uruguay (45 km north of the town of Treinta y Tres)”.

#### Paratypes examined

FMNH 10564–6, MZUSP 6483 (ex FMNH 10497), adult males from the type locality.

#### Additional adult and juvenile specimens examined

One hundred and eight specimens from several localities in the Brazilian State of Rio Grande do Sul, and 44 specimens from many localities in the Uruguayan departments of Cerro Largo, Maldonado, Rivera, Rocha, Tacuarembó, and Treinta y Tres. See S1 Appendix for account.

#### Diagnosis

*Scinax uruguayus* can be differentiated from *S*. *fontanarrosai* sp. n. and *S*. *pinima* by the combination of (1) small size in females (SVL 23.8–27.6 mm; *n* = 6); (2) head broadly rounded in dorsal view (Fig 3A); (3) absence of interorbital grooves; (4) anterior portion of the choanae concealed by the palatal shelf of the maxillary arch when roof of mouth is viewed from below; (5) subtriangular cephalic blotch (Fig 3A); (6) bicolored iris with an iridescent golden upper half and a golden lower half with brown reticulations (Fig 3B); (7) discs of the fingers and toes golden yellow to orange in life (Fig 3C); (8) hidden surfaces of thighs and tibia light purple in life (Fig 3D); (9) frontoparietals as slender strips at the level of a completely exposed fontanelle; (10) thick, rectangular dentigerous process with 3–4 teeth; (11) palatines overlapping about 3/4 of planum antorbitale; (12) intercalary elements between ultimate and penultimate completely mineralized; (13) larynx with oval arytenoids, without a slightly medial constriction in dorsal view; (14) advertisement call composed of a single, short (17–28 ms), and pulsed note (7–10 pulses/note), emitted at a rate of 3.7–5.0 notes/s; (15) pulse rate of 286–476 pulses/s; (16) notes with pulses of decreasing amplitude modulation from the first to the last one; (17) notes lacking harmonic structure; and (18) dominant frequency between 3833–4651 Hz.

#### Description of holotype

See Schmidt [15] for a morphological description of the holotype.

#### Coloration in life of the holotype

See Schmidt [15] for a brief description of the coloration in life of the holotype.

#### Coloration in preservative of the holotype

The holotype of *Scinax uruguayus* is well preserved but quite discolored, with faded dorsal pattern. A whitish subtriangular cephalic blotch and light rounded spots are visible in its light brown dorsum; and the vocal sac is brown colored.

#### Variation and morphological observations on the adult specimens examined

**Fig 13:**
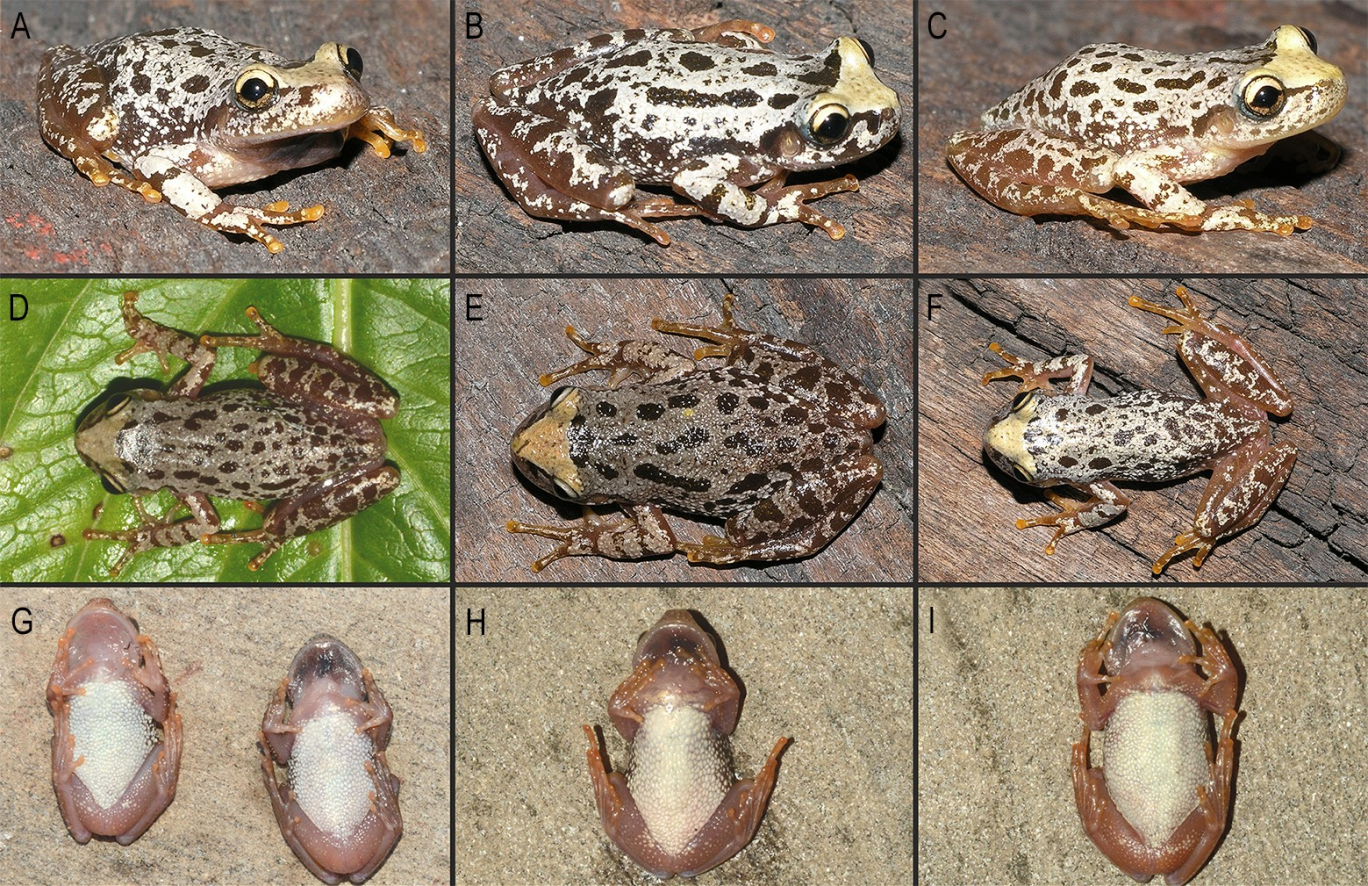
Color pattern of *Scinax uruguayus* in life. (A–C) Dorsolateral, (D–F) dorsal, and (G–I) ventral views.

Measurements of some adult specimens are presented in Table 1. The dorsal coloration pattern in life is somewhat variable in specimens of *Scinax uruguayus* (Fig 13). A light brown to gold subtriangular cephalic blotch is present in the region from the nostrils to the middle of the upper eyelids. The subtriangular blotch is laterally bordered by a thin dark brown line that extends from the nostrils to the eyes; this line can be posteriorly and anteriorly enlarged in some specimens. A fine light cream line along the posterior border of the cephalic blotch also can be present.

The dorsum is gray with scattered irregular, small, dark brown blotches. Irregular brown blotches are present at the border of the maxilla. An irregular, small, dark blotch in the posterior portion of the nostril can be present in some specimens. The dorsal surface of arms is light golden, with transversal dark blotches; dorsal surface of hindlimbs is grayish to dark brown, with irregular light golden blotches. The dorsal blotches can merge forming longitudinal irregular stripes and a transversal dark band adjacent to the subtriangular cephalic blotch in some individuals. Blotches might also form a fine reticulated pattern on the flanks.

Hidden surfaces of thighs and tibia are light purple. Pectoral and abdominal regions white. Ventral region of arms grayish. Black horizontal elliptical pupil; bicolored iris, with an iridescent golden upper half and a golden lower half with brown reticulations.

Males have quite expanded, dark brown or black colored, vocal sacs with longitudinal folds evident in deflated position (Fig 13G–13I), and light-colored nuptial pads without epidermal projections macroscopically evident. Pectoral fold with only a preaxillar element. The discs in hand and toes are rounded. Hand webbing basal. Toe webbing formula I 2^+^–2^+^ II 2^-^–3^+^ III 3^+^–2^-^ IV 3^+^–2^-^ V. The available females (SVL 23.8–27.6 mm; *n* = 6) have a size similar to that of the males (SVL 22.2–27.0 mm; *n* = 84).

#### Adult skeletal morphology

The skeletal morphology of *Scinax uruguayus* (LGE 4569, 4578–81) shares several characteristics with those of *S*. *fontanarrosai* sp. n. (LGE 2040, 4486–90) and *S*. *pinima* (UFMG 20185). We only point here the differences observed in *S*. *uruguayus* with respect to these species. (1) Nasals L-shaped (nasals narrow in *S*. *pinima*), with inner margins weakly overlapping the sphenethmoid posteriorly (non-overlapping in *S*. *pinima*). (2) Sphenethmoid with the anterior portion incorporating at least half of the *septum nasi* length, laterally extending to the level of, but not including the anterior process of the postnasal wall; and posteriorly covering the anterior margin of the frontoparietal fontanelle, at the level of the orbitonasal foramen (sphenethmoid with the anterior portion incorporates only the base of the *septum nasi*, not laterally extended, and posteriorly covering slightly the anterior margin of the frontoparietal fontanelle in *S*. *pinima*). (3) Frontoparietals as slender strips at the level of the fontanelle resulting in a fontanelle almost completely exposed (inner margins of the frontoparietals juxtaposed or slightly separated; the fontanelle is almost completely concealed in *S*. *fontanarrosai* sp. n. Fig 6A). (4) Prootics with a *crista parotica* poorly ossified laterad (with non-ossified laterad in *S*. *pinima*). (5) Maxilla posteriorly overlapping approximately half of the quadratojugal length (overlapping approximately one third of the quadratojugal length in *S*. *pinima*). (6) Anterior process of the vomers present, and prechoanal and postchoanal processes developed, limiting the medial margin of the choanas (anterior process of the vomers absent; prechoanal and postchoanal processes poorly developed, embracing a small portion of the medial margin of the choanas in *S*. *pinima*; Fig 6B). (7) Dentigerous process of the vomers represented by a thick, rectangular structure, with 3–4 pedicellate teeth (laminar without teeth in *S*. *fontanarrosai* sp. n., and pointed without teeth in *S*. *pinima*). (8) Palatines overlapping about 3/4 of planum antorbitale (palatines reduced to thin ossified slivers). (9) Posteromedial process of the parasphenoid absent (present, but extremely reduced in *S*. *fontanarrosai* sp. n.). (10) Arytenoids oval, without medial constriction in dorsal view (arytenoids oval, with a slight medial constriction in dorsal view in *S*. *fontanarrosai* sp. n.). (11) Intercalary elements between ultimate and penultimate phalanges completely mineralized (partially mineralized).

We also observed that some characters in *Scinax uruguayus* vary intraspecifically in *S*. *fontanarrosai* sp. n. (character states in parentheses). These are as follows. (1) Parasphenoid with a pointed cultriform process; anterior end is posterior to the orbitonasal foramen (rounded cultriform process; anterior end extending to the level of the orbitonasal foramen in some specimens of *S*. *fontanarrosai* sp. n.). (2) Prootics separated by a strip of cartilage medially (prootics medially fused in some specimens of *S*. *fontanarrosai* sp. n.). (3) Maxilla posteriorly surpassing the anterior margin of the tympanum (maxilla posteriorly reaching the anterior margin of the tympanum in some specimens of *S*. *fontanarrosai* sp. n.). (4) Hyoid plate slightly mineralized posteromedially, and two times wider than longer (entirely cartilaginous and one and a half times wider than longer in some specimens of *S*. *fontanarrosai* sp. n.). (5) Transverse process inclined posteriorly to body axis in the Presacral Vertebra VI (perpendicular in some specimens of *S*. *fontanarrosai* sp. n.).

Finally, there are some characters that vary intraspecifically in *Scinax uruguayus* but showed no variation in the skeletons of *S*. *fontanarrosai* sp. n. and/or *S*. *pinima*. These are as follows (character states of *S*. *fontanarrosai* sp. n. and *S*. *pinima* in parentheses). (1) *Tectum synoticum* entirely (LGE 4569, 4578) or partially ossified (LGE 4579, 4580) (*tectum synoticum* entirely cartilaginous in *S*. *pinima*). (2) *Pars dentalis* of premaxilla and maxilla with 8–15 and 33–36 pedicellate teeth, respectively (12–17 and 25–34 pedicellate teeth, respectively). (3) Dentigerous process continuous to the main body of vomers (LGE 4569, 4580) or contiguous to the main body by a medial, constricted portion (LGE 4578) (separated from the main body of vomers in some specimens of *S*. *fontanarrosai* sp. n.). (4) Median prenasal process of the *septum nasi* at the level of (LGE 4569, 4578, 4580) or surpassing (LGE 4579) the alary process of the premaxilla (surpassing in *S*. *fontanarrosai* sp. n. and at the level in *S*. *pinima*), and rounded shaped in dorsal view (triangular in some specimens *S*. *fontanarrosai* sp. n.). (5) Sternum distally simple (LGE 4579) or bilobed (LGE 4569, 4578, 4580) (simple in *S*. *pinima*). (6) Posteromedial process on the posterior margin of the sacral diapophysis absent (LGE 4581) or poorly developed (LGE 4569, 4578–80) (absent in *S*. *pinima* and absent, poorly developed or developed in *S*. *fontanarrosai* sp. n.).

#### Tadpoles

See Kolenc et al. [6] for a detailed description of the external morphology of the tadpole of *Scinax uruguayus* (Fig 7C). Measurements from 15 tadpoles at stages 31–33 (Lots ZVCB 10235, 10237, and 10240) are presented in Table 2.

#### Buccopharyngeal morphology of tadpoles

See Alcalde et al. [53] for a description of the buccopharyngeal morphology and musculoskeletal system of the tadpole of *Scinax uruguayus*.

#### Advertisement call

We reanalyzed calls (LGE-B 55) previously published for one topotype specimen (ZVCB 8224) of *S*. *uruguayus* [6]. In addition, we analyzed advertisement calls (LGE-B 56–67) of 12 specimens from three Uruguayan populations: five topotypic specimens (MNHN 9870–4), six specimens (MNHN 9875–80) from Valentines, Departamento de Treinta y Tres, and one specimen (MNHN 9881) from Laguna de Rocha, Departamento de Rocha. The advertisementcall of this species was described for specimens from Brazil [17] and Uruguay [6]. Our analyses of the advertisement calls of 13 specimens from Uruguayan populations showed that the advertisement call of *S*. *uruguayus* consists of a single, pulsed note, emitted at a rate of 3.7–5.0 notes/s (Fig 8C; Table 3). The note lasts 17–28 ms and the interval between notes is 157–290 ms. Each note has 7–10 pulses that are decreasingly modulated from the first to the last pulse within the note. Pulse rate of 286–476 pulses/s. Dominant frequency ranges from 3833–4651 Hz (Fig 8C).

#### Cytogenetics

The karyotype and banding patterns (Ag-NORs and C-band) of *Scinax uruguayus* were described by Cardozo et al. [103] (see Table 4).

#### Geographic distribution

*Scinax uruguayus* inhabits mostly the southern and southeastern portions of the Uruguayan Savanna (5–500 m a.s.l.), associated mainly to hilly environments, although some Uruguayan populations are present in lowland grasslands of the Laguna Merin basin (Treinta y Tres) and coastal areas close to the Atlantic Ocean at Laguna de Rocha. In addition, it is known from a few localities in the southeastern limits of the altitude grasslands (700–900 m a.s.l.) in the Araucaria Moist Forest Ecoregion, in the Brazilian State of Rio Grande do Sul (Fig 10).

#### Natural history

Detailed notes on the natural history of *Scinax uruguayus*, including data on its phenology, breeding sites, and clutch structure, were provided by Kolenc et al. [6]. We observed the passive defensive “crouching down” behavior in one juvenile of *S*. *uruguayus* (unvouchered specimen; Fig 11F). The amplexus is axillary and one female kept in captivity laid clutches with 398 eggs (single eggs sensu [70]) in water, attached to the aquatic vegetation ([6]; Fig 11G and 11H), and positioned singly or in irregular masses [6]. The ovum has a dark brown to black animal pole and a cream vegetal pole ([6]; Fig 11G), with a diameter of 1.3 ± 0.07 mm, and 1.62 ± 0.07 mm when the viteline membrane is considered; and the egg jelly diameter is 5.2 mm [6].

## Discussion

In a recent molecular phylogenetic study of Hylidae, limited to DNA sequences available in GenBank, Duellman et al. [9] erected the new genus *Julianus* for the species in the *Scinax uruguayus* group as defined by Faivovich et al. [3]. The genus was poorly defined, without any regard to synapomorphies proposed neither by earlier authors nor by themselves, based on few characteristics shared by most species included in Scinaxini. For example, the characters used to define *Julianus* are identical to those of *Scinax* [9]. Although ignored by Duellman et al. [9] and by Ferrão et al. [69], there are adult and larval morphological putative synapomorphies that diagnose the *Scinax uruguayus* group [2,3,6] as commented earlier in this study, and further discussed below.

We follow Colaço and Silva [107] in considering *Julianus* as a synonym of *Scinax*, as also done by Lourenço et al. [108], Conte et al. [109], Faivovich et al. [4], and Ron et al. [110]. Faivovich et al. [4] extensively discussed the problems regarding this and other optional taxonomic changes implemented by Duellman et al. [9].

### Synapomorphies of the *Scinax uruguayus* group

The monophyly of the *Scinax uruguayus* group has not been formally tested, since *S*. *pinima* (as *S*. *uruguayus*) was the only species included in previous phylogenetic analyses (e.g., [3,9,10]). However, four morphological synapomorphies were proposed for this group: (i) bicolored iris in adults, (ii) reduction of toe webbing, (iii) presence of two keratinized and dark colored plates on the sides of the lower jaw sheath, and (iv) marginal papillae on the posterior margin of the oral disc larger than those on the lateral margins [3,6]. They are discussed below.

(i) Adults of *Scinax fontanarrosai* sp. n., *S*. *pinima*, and *S*. *uruguayus* share a bicolored iris with a golden upper half and a dark brown to black or reticulated lower half (with or without golden chromatophores or brown reticulations in the lower half; Fig 3B), a somewhat unique character state in *Scinax*. A bicolored iris with a reddish upper half and a grey lower half was reported for *S*. *ruberoculatus* Ferrão, Fraga, Moravec, Kaefer, and Lima, 2018 so far (*S*. *ruber* clade; [111]). In Hylinae, eyes with bicolored iris—with different coloration pattern—have also been observed for adults of *Osteocephalus leoniae* Jungfer and Lehr, 2001 (Lophyohylini; [112]) and all species of *Aplastodiscus* (Cophomantini; [113]).

(ii) The toe webbing is reduced between toes II and V in the species of the *Scinax uruguayus* group with respect to remaining species of *Scinax*. However, the interspecific variation in the degree of webbing is relatively continuous and difficult to partition into discrete states; exceptions are the webbing between toes II and III, and the preaxial and postaxial webbing of Toe IV (Figs 2D and 3C). The extreme reduction of webbing between toes II and III—the webbing reaches the distal margin of the subarticular tubercle of Toe II—occurs in species of the *S*. *uruguayus* and *S*. *perpusillus* groups and was considered a synapomorphy of the latter [2,114]. While the webbing reaches the midlength of the first phalanx of Toe II (e.g., *S*. *alter* (Lutz, 1973), *S*. *canastrensis* (Cardoso and Haddad, 1982), *S*. *haddadorum* Araujo-Vieira, Valdujo, and Faivovich, 2016, and *S*. *humilis* (Lutz and Lutz, 1954); [115–118]) or the base of the disc in the remaining species of *Scinax* (e.g., *S*. *eurydice* (Bokermann, 1968), *S*. *hayii* (Barbour, 1909), and *S*. *perereca* Pombal, Haddad, and Kasahara, 1995; [20,119,120]) and related genera (e.g., *Dendropsophus*, *Pseudis*, *Sphaenorhynchus*, and *Scarthyla*; [121–124]). Therefore, under the recent phylogenetic hypotheses proposed for Hylidae (e.g., [9,10]) this character state could be a putative synapomorphy of both species groups.

*Scinax fontanarrosai* sp. n., *S*. *pinima*, and *S*. *uruguayus* share reduced preaxial and postaxial webbing of Toe IV—webbing reaches the proximal half of antepenultimate phalanx in Toe IV—with some species of the *S*. *ruber* clade (e.g., *S*. *cabralensis* Drummond, Baêta, and Pires, 2007, *S*. *caldarum* (Lutz, 1968), *S*. *duartei* (Lutz, 1951), *S*. *maracaya* (Cardoso and Sazima, 1980), *S*. *squalirostris*, and *S*. *villasboasi* Brusquetti, Jansen, Barrio-Amorós, Segalla, and Haddad, 2014; [118,125–130]), and several species of the *S*. *catharinae* clade (e.g., species in the *S*. *perpusillus* group; *S*. *ariadne* (Bokermann, 1967), *S*. *carnevallii* (Caramaschi and Kisteumacher, 1989), *S*. *centralis* Pombal and Bastos, 1996, *S*. *heyeri* (Peixoto and Weygoldt, 1986), and *S*. *humilis*; [114,115,131–134]). Other species of *Scinax* and other genera have more extensive webbing that reaches the proximal half of the penultimate phalanx in Toe IV (e.g., *S*. *canastrensis*, *S*. *catharinae* (Boulenger, 1888), *S*. *fuscovarius*, *S*. *haddadorum*, *S*. *rupestris* Araujo-Vieira, Brandão, and Faria, 2015, *Dendropsophus*, *Scarthyla*, *Sphaenorhynchus*, and *Xenohyla*; [118,121,123,124,135,136]) or the base of the disc (*Lysapsus*, *Pseudis*, and *Sphaenorhynchus lacteus* (Daudin, 1800); [122,137]). Under the recent phylogenetic hypotheses of Hylidae [9,10]—considering that the extent of webbing along each margin of the digit varies independently [138]—both reduced preaxial and postaxial webbing of Toe IV could be synapomorphies of the *S*. *uruguayus* group with some instances of homoplasy in *Scinax* (e.g., *S*. *perpusillus* group). However, these characters need to be tested on a comprehensive phylogenetic hypothesis of *Scinax* before being considered synapomorphies of this group.

(iii) Tadpoles of *Scinax fontanarrosai* sp. n., *S*. *pinima*, and *S*. *uruguayus* share the presence of two keratinized and dark colored plates on the sides of the lower jaw sheath ([3,6,53]; see also Fig 7). These keratinized plates also occur in tadpoles of *S*. *camposseabrai* (Bokermann, 1968) [139]. The phylogenetic position of *S*. *camposseabrai* within *Scinax* remains unstudied. This information is essential to understand the optimization of this character state in *Scinax*. In the meantime, we consider it as a putative synapomorphy of the *S*. *uruguayus* group, with at least one known instance of homoplasy in *S*. *camposseabrai* whithin Hylinae [3]. These plates have been also described in larvae of the hylids *Litoria peronii* (Tschudi, 1838) and *L*. *verreauxii* (Duméril, 1853) (Pelodryadinae; [140]) and distantly related groups of anurans such as *Kassina cassinoides* (Boulenger, 1903), *K*. *cochranae* (Loveridge, 1941), *K*. *decorata* (Angel, 1940), *K*. *kuvangensis* (Monard, 1937), *K*. *fusca* Schiøtz, 1967, *K*. *maculosa* (Sternfeld, 1917), *K*. *senegalensis* (Duméril and Bibron, 1841), *K*. *schioetzi* Rödel, Grafe, Rudolf, and Ernst, 2002, *Paracassina kounhiensis* (Mocquard, 1905), *P*. *obscura* (Boulenger, 1895), *Phlyctimantis boulengeri* Perret, 1986, *P*. *keithae* Schiøtz, 1975, *P*. *maculatus* (Duméril, 1853), *Semnodactylus wealii* (Boulenger, 1882) (Hyperoliidae), *Trichobatrachus robustus* Boulenger, 1900 (Arthroleptidae), and species of the *Lithobates pipiens* group (Ranidae) [36,141,142].

(iv) Enlarged marginal papillae on the posterior margin of the oral disc are present in larvae of *Scinax fontanarrosai* sp. n., *S*. *pinima*, and *S*. *uruguayus* ([6,53]; see also Fig 7). The presence of these papillae has also been reported in larvae of *S*. *camposseabrai* [139] and some species of *Sphaenorhynchus* [123,137]. We consider the presence of marginal papillae on the posterior margin of the oral disc larger than those on the lateral margins as a putative synapomorphy of the *S*. *uruguayus* group, with at least two instances of homoplasy in Hylinae—in *S*. *camposseabrai* and in an internal clade of *Sphaenorhynchus* (see [137]).

Apart from the six character states mentioned above (characters i, iii, and iv plus three independent characters from the former character ii), there are some morphological characters that could be considered synapomorphies of the *Scinax uruguayus* group or of a less inclusive clade. The presence of a subtriangular or V-shaped cephalic blotch in the *S*. *uruguayus* group (subtriangular in *S*. *uruguayus*; and V-shaped in *S*. *fontanarrosai* sp. n. and *S*. *pinima*; Figs 3A, 4, 12 and 13) could be another possible synapomorphy of this group. No other species of *Scinax* or related genera (*Dendropsophus*, *Lysapsus*, *Pseudis*, *Scarthyla*, *Sphaenorhynchus*, and *Xenohyla*; [136,143,144]) have a cephalic blotch alike (subtriangular or V-shaped). Some species in the *S*. *catharinae* clade have a light triangular blotch in the anterior portion of the head from the interorbital area to the tip of snout. However, unlike the color pattern in the *S*. *uruguayus* group, the triangle results from an interruption of the light dorsal pattern color of the body by a darker interocular triangular blotch and not a distinct coloration patch (e.g., *S*. *catharinae*, *S*. *centralis*, *S*. *hiemalis* (Haddad and Pombal, 1987), *S*. *rizibilis* (Bokermann, 1964), *S*. *trapicheiroi* (Lutz and Lutz, 1954); [115,134, 145,146]). The presence of dermal interorbital grooves in *S*. *fontanarrosai* sp. n. and *S*. *pinima* (absent in *S*. *uruguayus*; Fig 14 in [5], Fig 26 in [13]; see also Figs 3A and 13) could be a putative synapomorphy of *S*. *fontanarrosai* sp. n. and *S*. *pinima*. These interorbital grooves are known so far only in these species among hylines. The histological structure of the interorbital grooves might correspond to connective tissue trabeculae between the subjacent epymisium and the hypodermis (C Taboada, personal communication), but more studies are required to corroborate this.

Studies on skeletal morphology of species of *Scinax* are scarce, limited to a few comparative studies (*S*. *acuminatus* (Cope, 1862), *S*. *fuscovarius*, *S*. *nasicus* (Cope, 1862), and *S*. aff. *ruber*; [147,148]) and some characters used in phylogenetic analyses [2,149]. Two novel osteological character states were observed by us in *S*. *fontanarrosai* sp. n., *S*. *pinima*, and *S*. *uruguayus*: the short medial ramus of pterygoid (concealing less than half of the anterior face of the basal process; Fig 6C) and expanded iliosacral sesamoids (at least two times longer than their longitudinal axis; Fig 6D). The short medial ramus of the pterygoid present in the *S*. *uruguayus* group might be unique within *Scinax*, since other species in this genus have an elongate medial ramus that covers the basal process but does not contact the prootic [2,149] (K Araujo-Vieira and J Faivovich, personal observations). An elongated medial ramus of pterygoid was reported in species of other hylid genera that had been related to *Scinax* (*Dendropsophus*, *Lysapsus*, *Pseudis*, *Scarthyla*, and *Sphaenorhynchus*; [21,136,137,149,150]). The only known exception is *Xenohyla truncata* (Izecksohn, 1959) (Fig 2 in [144]) that has a short medial ramus similar to that present in species of the *S*. *uruguayus* group. The short medial ramus of pterygoid could be another synapomorphy of this group.

The round or trapezoid iliosacral sesamoids occur in some species of *Scinax* (Fig 14 in [147]), whereas a medially expanded iliosacral sesamoid—the transversal axis at least two times longer than the longitudinal axis—is present in *S*. *fontanarrosai* sp. n., *S*. *pinima*, and *S*. *uruguayus*. While a round or trapezoid sesamoid might be present in all species of *Scinax* (K Araujo-Vieira and J Faivovich, personal observations), the medially expanded sacral sesamoid seems to be unique among anurans. Other hylids have an anteroposteriorly elongated sacral sesamoid, with the longitudinal axis at least two times longer than the transversal axis (Fig 14 in [147], [151,152]). The shape and size of the sacral sesamoids is one of the elements (the other being degree of sacral expansion, the iliolumbaris muscle, and the articular ligament; [147,153]) that determine the direction of movement at the iliosacral articulation of a frog. A medially expanded sacral sesamoid may provide greater lateral and vertical rotation capability to the iliosacral joint. However, the origin and insertion of the articular ligaments are also decisive for the functional interpretation of the patterns of iliosacral articulation [147,153]. Histological studies on the iliosacral joint and functional analyses are necessary to better understand the role of the sesamoids in the iliosacral articulation in *Scinax*. Meanwhile, we suggest that the medially expanded iliosacral sesamoid is a putative synapomorphy of the *S*. *uruguayus* group.

Tadpoles of the *Scinax uruguayus* group have the intestinal coiling axis subparallel to the main body axis (Character 78 in [2], [16]). This character state was previously suggested as a synapomorphy of the clade including *S*. *acuminatus* + the *S*. *rostratus* group by Faivovich [2]. This character has an ambiguous optimization in a phylogenetic framework in which *S*. *uruguayus* is recovered as the sister taxon of the clade including *S*. *acuminatus* + the *S*. *rostratus* group plus the remaining species of the *S*. *ruber* clade [3,7–10]. The intestinal coiling axis subparallel to the main body axis would have arisen in the ancestor of the *S*. *ruber* clade with a subsequent reversion in the sister taxon of *S*. *acuminatus* + the *S*. *rostratus* group. Alternatively, it may have evolved independently in the *S*. *uruguayus* group and the clade including *S*. *acuminatus* + the *S*. *rostratus* group.

Considering our observations and the putative synapomorphies mentioned above, the *Scinax uruguayus* group (combined SVL, males 19.1–27.0 mm, females 23.8–29.0 mm; [5]) can be diagnosed by the following phenotypic characters: (1) presence of pectoral fold (only the preaxillar element); (2) light-colored nuptial pads without macroscopically evident epidermal projections; (3) round discs in hand and toes; (4) reduced webbing between toes II and II (the webbing reaches the distal margin of the subarticular tubercle of Toe II); (5) reduced preaxial webbing in Toe IV (webbings reach the proximal half of antepenultimate phalanx in Toe IV); (6) reduced postaxial webbing in Toe IV (webbings reach the proximal half of antepenultimate phalanx in Toe IV); (7) bicolored iris (golden upper half and dark brown to black or golden lower half) in adults (Fig 3B); (8) subtriangular or V-shaped cephalic blotch (Fig 3A); (9) dark-colored, externally expanded vocal sac, evident by the presence of loose skin with numerous longitudinal folds (Figs 4, 12 and 13); (10) short medial ramus of pterygoid, concealing less than half of the anterior face of the basal process (Fig 6C); (11) presence of anterior process of the suprascapula; (12) medially expanded iliosacral sesamoids, at least two times longer than their longitudinal axis (Fig 6D); (13) tadpoles with marginal papillae on the posterior margin of the oral disc larger than those on the lateral margins; (14) dark colored keratinized plates on the sides of the lower jaw sheath; (15) well-developed, higher than wide, conspicuously keratinized jaw sheaths (Fig 7); and (16) intestinal coiling axis subparallel to the main body axis. The character states 4–8, 10, and 12–14 are putative synapomorphies of this group.

### Buccopharyngeal and musculoskeletal morphology of tadpoles

Larvae of *Scinax fontanarrosai* sp. n. (as *S*. aff. *pinima*; [53]), *S*. *pinima* (this work), and *S*. *uruguayus* [53] have distinctive traits in the oral cavity that differentiate them from other larvae of *Scinax* that have been studied (see Appendix I in [53]). They are multifid and overlapping infralabial papillae, large lateral ridge papillae, and well defined buccal floor arena and ventral velum. Some musculoskeletal features seem to be also characteristic of *S*. *fontanarrosai* sp. n. and *S*. *uruguayus*; e.g., cornua trabeculae with short and wide free portions, processus articularis short and wide, processus muscularis narrow and directed anteriorly, cartilago suprarostralis forming a single structure, lower jaw cartilages massive, ceratobranchialia II–IV continuous with the planum hypobranchiale, and m. *subarcualis* rectus I formed by two slips [53]. The buccopharyngeal morphology, chondrocranium, and cranium muscles remain poorly studied in tadpoles of *Scinax* (see Appendix I in [53]). The taxonomic distribution of these character states requires a more extensive sampling to assess their polarity.

### Advertisement calls

The advertisement call of *Scinax uruguayus* was described by Kwet [17], based on a single individual from the north of the State of Rio Grande do Sul, Brazil. Kolenc et al. [6] further described the advertisement call of one individual from Department of Lavalleja, Uruguay with some differences (number of pulses, interval between notes they regarded them as mainly produced by the different analytical methods employed). Other differences (duration of the set of notes, note rate, dominant frequency) could be associated to variables such as temperature, size, and/or degree of excitation of the specimens [154]. Our analyses of *S*. *uruguayus* (calls of 13 individuals from three localities), including a topotype, encompass the range values of the spectral and temporal parameters provided by Kolenc et al. [6] (Fig 8C; see also Table 3). Furthermore, the number of pulses per note (7–10 pulses/note) was exactly the same, and broadly overlap with the range (6–9 pulses/note) resulting from the analyses of calls by Kwet [17].

The advertisement call of *Scinax pinima* was described by Bokermann and Sazima [5]. Some parameters of the original description differ from our results. For instance, our analyses showed calls with note rates of 3.0–3.3 notes/s *vs*.8–9 notes/s reported by these authors. We noticed that 8–9 notes/s is not congruent with note duration of 0.1 s and interval between notes of 0.2 s already reported. Bokermann and Sazima [5] also reported a harmonic structure for the call of *S*. *pinima*, not seen in our analyses of this species. The poorly differentiated harmonics described by these authors seem to be sidebands, a phenomenon resulting of high pulse rates [155].

The advertisement call of *Scinax pinima* seems to be fairly constant along its wide latitudinal distribution (Fig 8B; see also Table 3). Temporal and spectral parameters did not show significant differences between calls recorded at the type locality (Serra do Cipó, Minas Gerais) and Lebón Regis (Santa Catarina, ~1100 km from the type locality). Moreover, the pulse amplitude modulation of the notes, with the first pulse of each note being distinctly lower than the second one, is also present in calls from both populations. Although with similar pulse amplitude modulation, call notes of *S*. *uruguayus* and *S*. *pinima* can be promptly distinguished by the presence of a lower first pulse in the latter.

*Scinax fontanarrosai* sp. n. has the most distinctive advertisement call when compared to those of *S*. *pinima* and *S*. *uruguayus*. It has higher values of note duration, number of pulses per note, pulse rate, and dominant frequency than those of *S*. *pinima* and *S*. *uruguayus* (Fig 8; see also Table 3). Values of pulse rate and dominant frequency overlap between *S*. *pinima* and *S*. *uruguayus* but not between these and the new species. The advertisement call of *S*. *fontanarrosai* sp. n. is unique in the *S*. *uruguayus* group for having notes with pulses forming an elliptical shape on the oscillograms. This is a result of pulses that are increasingly modulated for the first quarter of the note, remaining with relatively constant amplitude in the second quarter and then decreasing up to the end of the note. Additionally, the occurrence of harmonics in the advertisement call of *S*. *fontanarrosai* sp. n. distinguishes it from *S*. *pinima* and *S*. *uruguayus* (absent in these species). The presence of harmonic structure in the advertisement calls of *Scinax* was previously suggested as being restricted to the *S*. *catharinae* group [120,156]. However, this is well known for many species of the *S*. *ruber* clade (e.g., [157,158]).

The species of the *Scinax uruguayus* group have high-pitched advertisement calls characterized by elevated dominant frequencies. The vocalizations of *S*. *fontanarrosai* sp. n. has the highest Dominant Frequency (DF) recorded for the *S*. *ruber* clade (5513–6159 Hz), whereas *S*. *uruguayus* (3833–4651 Hz) and *S*. *pinima* (3919–4479 Hz) have DF comparable only to some small-sized species of this clade, yet unassigned to any group: *S*. *fuscomarginatus* (Lutz, 1925) (2928–5383 Hz; [120,130,159–161]); *S*. *madeirae* (Bokermann, 1964) (3100–5672 Hz; [130,161]); *S*. *altae* (Dunn, 1933) (3379–4056 Hz; [162]); *S*. *squalirostris* (3800–4600 Hz; [120,163,164]); *S*. *staufferi* (Cope, 1865) (3950–4350 Hz; [165]); *S*. *tymbamirim* Nunes, Kwet, and Pombal, 2012 (4000–4300 Hz; [117]); *S*. *exiguus* (Duellman, 1986) (4000–4800; [159]); *S*. *auratus* (Wied-Neuwied, 1821) (4000–4350 Hz; [166]); *S*. *baumgardneri* (Rivero, 1961) (4100–4600 Hz; [167]); *S*. *cardosoi* (Carvalho-e-Silva and Peixoto, 1991) (3281–4828 Hz; [168]); and *S*. *wandae* (Pyburn and Fouquette, 1971) (4800–5050 Hz; [159,169]).

### Cytogenetic data

The karyotype of *Scinax fontanarrosai* sp. n. resembles that of *S*. *uruguayus* ([103]; Fig 9). Like other species of the *S*. *ruber* clade, the chromosome Pair 1 is metacentric in *S*. *fontanarrosai* sp. n. and *S*. *uruguayus*, whereas it is submetacentric and significantly smaller in species of the *S*. *catharinae* clade (e.g., [103,170–179]). Cardozo et al. [103] suggested that the submetacentric Pair 1 constitutes a synapomorphy of the *S*. *catharinae* clade. This feature is shared also in the karyotypes of *Dendropsophus* (2*n* = 30) but probably Pair 1 of this genus is not homologous with that of the *S*. *catharinae* clade [103,180].

Cardozo et al. [103] pointed also that the differences in size and morphology of the chromosome Pair 1 between species of the *Scinax catharinae* and *S*. *ruber* clades could be attributed to pericentric inversions, addition/loss of repetitive sequences, or still unidentified structural chromosome alterations. Gruber et al. [176], based on replication bands, attributed these differences to the loss of repetitive sequences in the chromosomes Pair 1 in two species of the *S*. *catharinae* clade (*S*. *littoralis* (Pombal and Gordo, 1991) and *S*. *hiemalis*). However, their results were not conclusive and new approaches to the molecular cytogenetic of *Scinax* would help to elucidate the underlying mechanisms.

*Scinax fontanarrosai* sp. n., *S*. *uruguayus*, and most other species of the *S*. *ruber* clade have NORs on Pair 11 (e.g., [103,172–175,179]), like many other Hylines with 2*n* = 24 (see [177,178], and cites therein). The exceptions are *S*. *alter* (on Pair 3; [103]), *S*. *boesemani* (Goin, 1966) (on Pair 8; [172]), and *S*. *constrictus* Lima, Bastos, and Giaretta, 2005 (on Pair 1; [179]). Nevertheless, most species of the *S*. *catharinae* clade have NORs in the Pair 6p; [103]; the two exceptions are *S*. *centralis*, with NORs on Pair 1q [179], *S*. *canastrensis*, which NORs are in both pairs 6 (in one female) and 11 (in males). Cardozo et al. [103] suggested the NOR placed in the short arm of the chromosome Pair 6 is a putative synapomorphy of the *S*. *catharinae* clade.

### Conservation

*Scinax fontanarrosai* sp. n., *S*. *pinima*, and *S*. *uruguayus* exclusively inhabit open grassland areas and savanna environments (i.e., Uruguayan Savanna, Southern Cone Mesopotamian Savanna, Araucaria Moist Forests, and Campos Rupestres Montane Savanna; Fig 10). These Ecoregions were formerly partially isolated in recent times by forested areas (i.e., Alto Paraná Atlantic Forest, Serra do Mar Coastal Forests, Cerrado, and Bahia Interior Forests), in which there are no records of these species. This disjunct pattern caught the attention of many authors (i.e., [6,13,16,17,96]). The latitudinal gap of ~900 km includes the entire State of São Paulo and southern Minas Gerais, which are one of the most densely sampled areas by Brazilian herpetologists [181]. A similar disjunct distribution pattern is observed for species of the *Proceratophrys bigibbosa* group, with *P*. *palustris* Giaretta and Sazima, 1993 known only from Minas Gerais and the remaining species (i.e., *P*. *avelinoi* Mercadal de Barrio and Barrio, 1993, *P*. *bigibbosa* (Peters, 1872), and *P*. *brauni* Kwet and Faivovich, 2001) occurring in the Brazilian states of Paraná, Santa Catarina, Rio Grande do Sul; northeast Argentina; and eastern Paraguay [182]. Extensive molecular studies (i.e., phylogenetic and phylogeographic) would allow understanding these intriguing convergent biogeographic patterns in South American frogs.

Populations of *Scinax fontanarrosai* sp. n. are dense, and seem to be tolerant to anthropogenic disturbances. For instance, we observed several specimens near national highways and in areas used for agriculture and extensive livestock production in Corrientes and Misiones, Argentina. Nevertheless, *S*. *pinima* was previously considered a microendemic species, whose range was restricted to the type locality and nearby areas in Serra do Cipó, Minas Gerais. Only three specimens (in 1987, 1992, and 2016; J Pombal Jr., personal communication; CFB Haddad, personal observation) were collected at the type locality after the earlier 1970´s [5]. The scarce biological information and the apparent small population size lead researchers to consider the conservation status of *S*. *pinima* as “Data Deficient” in IUCN Red List [183]. However, we identified several populations of *S*. *pinima* farther south, previously reported as *S*. *uruguayus* (or *Hyla uruguaya*) from the Brazilian states of Paraná, Santa Catarina, and Rio Grande do Sul (e.g., [11,17,18]; see also Fig 10). Therefore, based on our new observations about geographic distribution, conspicuousness, and habitats of occurrence, we suggest including *S*. *fontanarrosai* sp. n. and *S*. *pinima* in the “Least Concern” category of the IUCN Red List. Despite this, it is necessary to study the status of the northernmost population of *S*. *pinima* to better understand the dynamics of its fluctuation, and if it entails any risk of local extinction. In regard to *S*. *uruguayus*, although considered rare by some authors [78,84] it is a fairly abundant species ([6]; this work) and we recommend inclusion in the “Least Concern” category of the IUCN Red List.

## Supporting information

S1 Appendix Studied specimens. Institutional abbreviations follow Sabaj [19]. AA: Cleared and double stained specimen.
(DOCX)
