# Correction: A review of the elusive bicolored iris Snouted Treefrogs (Anura: Hylidae:*Scinax uruguayus* group)

**DOI:** 10.1371/journal.pone.0225543

**Published:** 2019-11-14

**Authors:** Diego Baldo, Katyuscia Araujo-Vieira, Dario Cardozo, Claudio Borteiro, Fernando Leal, Martín O. Pereyra, Francisco Kolenc, Mariana L. Lyra, Paulo C. A. Garcia, Célio F. B. Haddad, Julián Faivovich

In Fig 8, panels B and C are incorrectly swapped. The authors have provided a corrected version here.

**Fig 8:**
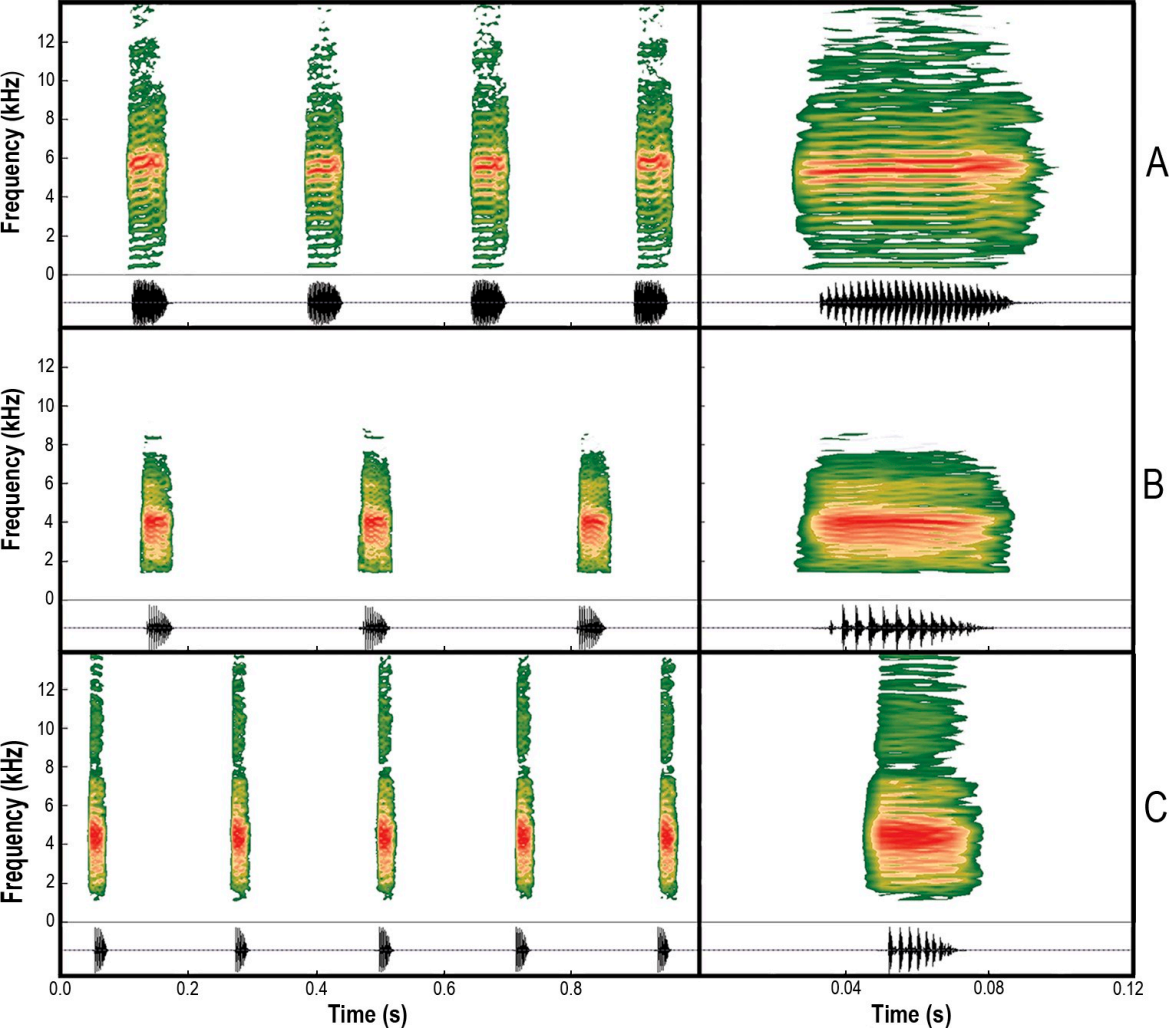
Audiospectrograms (above) and oscillograms (bellow) of the advertisement calls. (A) *Scinax fontanarrosai* sp. n. (LGE 4451), (B) *S*. *pinima* (UFMG 20184), and (C) *S*. *uruguayus* (MNHN 9877). Left: A series of calls. Right: A single call.
