# Supplementary material for: A review of the elusive bicolored iris Snouted Treefrogs (Anura: Hylidae:Scinax uruguayus group)
Source: PLoS One. 2019 Sep 25;14(9):e0222131. doi: 10.1371/journal.pone.0222131 (PMC6760762; doi:10.1371/journal.pone.0222131)
Supplement: S1 Appendix — Institutional abbreviations follow Sabaj [19]. AA: Cleared and double stained specimen. (DOCX) [file pone.0222131.s001.docx]

**Supporting information**

**S1 Appendix. Studied specimens**. Institutional abbreviations follow Sabaj [19]. AA: Cleared and double stained specimen.

*Scinax pinima*.—**BRAZIL**: Minas Gerais: Santana do Riacho, Alto Palácio, Serra do Cipó, km 132, MZUSP 73668 (ex WCAB 46238; holotype), MZUSP 73859–63 (ex WCAB 47439–40, 47442–3, 47445; paratypes), UFMG 20179, 20181–4, 20185 (AA), UFMG 2262 (tadpoles); Santana do Riacho, Alto Palácio, CFBH 35054, 39978; Estrada de Vespasiano a Conceição do Mato Dentro km 110–132, CFBH 6241–2 (ex WCAB 47429–30, paratypes). Paraná: General Carneiro, Indústria Pedro Pizzatto, CFBH-T 11197, 11209. Rio Grande do Sul: Cambará do Sul, CFBH 5788, MCP 0329; Cambará do Sul, Parque Nacional de Aparados da Serra, MCP 3963–5. Santa Catarina: Campo Belo do Sul, Área AC3 de monitoramento da UHE Barra Grande, UFRGS 04055–6; Campo Belo do Sul, Área AD2 de monitoramento da UHE Barra Grande, UFRGS 4115; Campos Novos, LGE 10598 (ex MCP 00528), 10600, MCP 00523–6, 00529–31; Campos Novos, AHE, MCP 9428–31; Campos Novos, Fazenda Manfroi, MCP 9078; Lages, Coxilha Rica, Fazenda Sobradinho, MCP 10385; Lebon Régis, Fazenda Serra da Esperança, LGE 21256; Palmas, ZUEC 11551, 11536 (tadpoles).

*Scinax uruguayus.*—**BRAZIL**: Rio Grande do Sul: Bagé, Área de Monitoramento do Parque Eólico Santa Tecla, UFRGS 05708, 05711, 05713, 05717, 05785–6; Caçapava do Sul, LGE 01786–7 (ex ZUEC 04157–8), MCN 04885, MCN 09328–50, 09352–3, 09374–80, 09407–13, MCP 09880; near Canion Fortaleza, MCP 03963–5; Candiota, MCP 04127, 04140–1, 04146, 04186, 05047, 05089, 05192; Caxias do Sul, MCP 10345; Criúva, MCP 12637–8; Lavras do sul, UFRGS 02642–3; Pinheiro Machado, UFRGS 05203, 05205; Área para implantação de Parque Eólico, Área da Fazenda 27, UFRGS 05796, 05807; Piratini, UFRGS 04925–9; Porto Alegre: Jardim Botânico, MCN 12613; Morro Teresópolis, MCN 02132; Santana do Livramento, Cerro Verde, UFRGS 05146–7; São Francisco de Paula: CFBH 03044–5; Potreiro Novo, LGE 10599 (ex MCP 03652), 10632 (ex MCP 02497), MCP 1804, 2498, 3207, 3410, 3434, 3649–51, 3653, 4959, 4504–5; Flona, MCN 13501–2; São Jerônimo, Fazenda Novos Ares, UFRGS 05875; São Sepé, CFBH 13142, MCP 11363–4, UFRGS 02669–70, 02674; Viamão, Águas Claras, Lombas, Estância dos Braun, MCN 12817–8, 12820–2, 13016. **URUGUAY**: Cerro Largo: Río Tacuarí, MNHN 1475; 20 km. NW from Plácido Rojas, MNHN 6215–7. Lavalleja: Arroyo Campanero Grande at National Route 8, MNHN 9882, 9888 (AA); Sierra de Minas, 12 km E from Minas, ZVCB 8224; Maldonado: Sierra de Carapé, 14 km SE from Aiguá, ZVCB 8222–3, ZVCB 10235 (tadpoles); Sierra de Carapé, 22 km SE from Aiguá, ZVCB 10236; Sierra de Carapé, 32 km SE from Aiguá, ZVCB 8225; Sierra de Carapé, Ruta 109, 18 km NW from Rocha, ZVCB 10237 (tadpoles); Rivera: Pueblo Madera, 10 km S from Rivera, ZVCB 10238–9, 10240 (tadpoles); Pueblo Madera, Ruta Nº 5, km 492, ZVCB 10612 (tadpoles); Rocha: La Pedrera, MNHN 9883; La Paloma, near Laguna de Rocha, MNHN 9881; Sierra de Carapé, Ruta 109, 41 km from Aiguá, MNHN 9885 (AA), ZVCB 8227–8; Tacuarembó: Gajo norte del Arroyo Tres Cruces, Sierras del Infiernillo, MNHN 1474, 3842–5. Treinta y Tres: Near to Quebrada de los Cuervos, ZVCB 10241–3; Puntas del Arroyo del Parao, 50 km N Ciudad de Treinta y Tres, MNHN 9870–4, 9889 (AA), ZVCB 8226; Quebrada de los Cuervos, ZVCB 14574–6, MNHN 9884 (tadpoles), MNHN 9886–7 (AA); 45 km north of town of Treinta y Tres, FMNH 10567 (holotype), FMNH 10564–6 (paratype), MZUSP 6483 (ex FMNH 10497; paratype); Ruta 8 and access to Quebrada de los Cuervos, LGE 4569 (AA); Sierra del Tigre, ZVCB 10244; Valentines, MNHN 9875–80.
